# Cytoprotective Effects of Lysophospholipids from Sea Cucumber *Holothuria atra*

**DOI:** 10.1371/journal.pone.0135701

**Published:** 2015-08-14

**Authors:** Yoshifumi Nishikawa, Ayumi Furukawa, Ikumi Shiga, Yoshikage Muroi, Toshiaki Ishii, Yayoi Hongo, Shunya Takahashi, Tatsuya Sugawara, Hiroyuki Koshino, Masao Ohnishi

**Affiliations:** 1 National Research Center for Protozoan Diseases, Obihiro University of Agriculture and Veterinary Medicine, Inada-cho, Obihiro, Hokkaido, Japan; 2 Department of Basic Veterinary Medicine, Obihiro University of Agriculture and Veterinary Medicine, Obihiro Hokkaido, Japan; 3 RIKEN Center for Sustainable Resource Science, Wako, Saitama, Japan; 4 Division of Applied Biosciences, Graduate School of Agriculture, Kyoto University, Kyoto, Japan; 5 Department of Food Science, Obihiro University of Agriculture and Veterinary Medicine, Obihiro, Hokkaido, Japan; University of British Columbia, CANADA

## Abstract

Lysophospholipids are important signaling molecules in animals and metazoan cells. They are widely distributed among marine invertebrates, where their physiological roles are unknown. Sea cucumbers produce unique lysophospholipids. In this study, two lysophospholipids were detected in *Holothuria atra* for the first time, lyso-platelet activating factor and lysophosphatidylcholine, with nuclear magnetic resonance and liquid chromatography–time-of-flight mass spectrometric analyses. The lipid fraction of *H*. *atra* contained lyso-platelet activating factor and lysophosphatidylcholine, and inhibited H_2_O_2_-induced apoptosis in the macrophage cell line J774A.1. The antioxidant activity of the lysophospholipid-containing lipid fraction of *H*. *atra* was confirmed with the oxygen radical absorbance capacity method. Our results suggest that the lysophospholipids from *H*. *atra* are potential therapeutic agents for the inflammation induced by oxidative stress.

## Introduction

Biologically active products from marine organisms have recently become the focus of pharmaceutical research and health food development. Among the aquatic invertebrates, sea cucumbers produce a diversity of secondary metabolites with valuable biological activities. The sea cucumber, *Holothuria*, which belongs to the phylum Echinodermata and the class Holothuroidea, is found on seafloors throughout the world [1]. It is considered a healthy food because it contains various physiologically active substances, including vitamins (A, C, B1, B2, and B3), trace elements (calcium, iron, magnesium, and zinc), polysaccharides (chondroitin sulfate), and saponin glycosides [2]. Sea cucumbers are also commonly used to treat wounds, eczema, arthritis, hypertension, and impotence [3].

Some bioactive compounds extracted from sea cucumbers are reported to have anti-inflammatory [4], antitumor [5], and fungicidal activities [6]. For instance, the sphingoid base composition of cerebrosides prepared from the sea cucumber is cytotoxic to human colon cancer cell lines [7]. Holotoxins from sea cucumbers are also well-known antifungal glycosides [8,9]. The characteristics of glycosphingolipids and their structure–activity relationships conferred neuritogenic activity against the rat pheochromocytoma cell line PC12 [10]. Further novel bioactive products may be discovered in the sea cucumber. In this study, we investigated the lipids of *Holothuria atra*, which is reported to have medicinal value [11]. A mixed extract of *H*. *atra* contained physiologically active phenolic compounds with antioxidant activity, which exerted potent hepatoprotective effects against thioacetamide-induced liver injury in a rat model [3]. Therefore, we undertook this study to identify the bioactive lipids in a mixed extract of the *H*. *atra* body wall and to evaluate their cytoprotective potential against oxidative stress. We found that lysophospholipids from *H*. *atra* inhibit H_2_O_2_-induced apoptosis in macrophages.

## Materials and Methods

### Ethics statement

Sea cucumbers were sampled after the appropriate permission was obtained from the Fishermen’s Cooperative Association in Okinawa Prefecture.

### Extraction and purification of lipids from *H*. *atra*

Sea cucumbers (*H*. *atra*) were collected from the ocean at Okinawa, Japan, and transported frozen on dry ice to our laboratory. The whole tissues (792.3 g) were homogenized in a blender with 1.5 L of chloroform–methanol (1:2, v/v) and mixed for 1 h at room temperature. The extract was filtered under reduced pressure to produce an aqueous suspension. The residue remaining after filtering was treated with 1.5 L of chloroform–methanol mixtures (1:4 and then 2:1, v/v) for 1 h each at room temperature, and the aqueous suspension was collected as described above. The aqueous suspension of the whole tissues was treated overnight with chloroform–methanol–water (8:4:3, v/v) using the Folch method. The lipid layer from the whole tissues was mixed to form the total lipid (TL).

The TL (8.965 g) was applied to a silica-gel column (Wakogel C-200, diameter 4 cm, height 40 cm, volume 502.4 cm^3^; Wako Pure Chemical, Osaka, Japan) in chloroform and eluted sequentially with chloroform–methanol (4:1, 2:1, 1:1, and 1:4, v/v), methanol, chloroform–methanol–water (3:6:1, v/v), and chloroform–water (4:1, v/v).

To detect the lipids, the TL was analyzed with silica gel thin-layer chromatography (TLC; silica gel 60, 0.25 mm; Merck, Darmstadt, Germany) with a chloroform–methanol–water mixture (65:25:4 or 65:35:8, v/v/v). The lipid species were visualized by spraying the plate with 50% sulfuric acid and heating it briefly. The carbohydrates and phospholipids were detected on the TLC plate with anthrone–sulfuric acid reagent and Dittmer reagent, respectively.

The fractionated lipids were evaporated with N_2_ and suspended in Tocrisolve 100 (Tocris BioScience, Bristol, UK) at 200 mg/mL.

### Reagents

Lyso-platelet activating factor (Lyso-PAF C18:0 and (9Z)-C18:1) were purchased from Avanti Polar Lipids (Alabaster, AL). Lysophosphatidylcholine (LPC, C18:0) and sphingomyelin were from Sigma (St Louis, MO).

### Measurement of cell viability

The mouse macrophage cell line J774A.1 (ATCC TIB-67) was cultured in Dulbecco’s modified Eagle’s medium (Sigma) supplemented with 10% heat-inactivated fetal bovine serum. The J774A.1 cells were seeded in 96-well flat-bottom plates (3.8 × 10^5^ cells/mL), incubated overnight at 37°C., and then preincubated with the lipids for 12 h. To induce apoptosis, the cells were treated with 112.5 μM or 25 μM H_2_O_2_. After 12 h, 2-(4-iodophenyl)-3-(4-nitrophenyl)-5-(2,4-disulfophenyl)-2*H*-tetrazolium monosodium salt and 1-methoxy-5-methylphenazinium methyl sulfate (Cell Counting Kit-8; Dojin Laboratories, Kumamoto, Japan) were added to final concentrations of 5 mM and 0.2 mM, respectively, and the cells were incubated for another 4 h. Proliferation was measured as the mean absorbance at 450 nm. Cell viability was expressed as the relative absorbance of the lipid-treated cells normalized to the absorbance of the untreated control cells (×100).

### Detection of apoptotic cells using terminal deoxynucleotidyl transferase (TdT)-mediated dUTP–biotin nick-end labeling (TUNEL)

To detect apoptosis directly, the cells were analyzed with TUNEL (*In Situ* Cell Death Detection Kit, TMR red; Roche Diagnostics GmbH, Mannheim, Germany). Briefly, the cells were placed on glass slides and dried. They were then fixed with 4% paraformaldehyde in phosphate-buffered saline (PBS) for 30 min at room temperature, washed in PBS, and permeabilized with 0.1% Triton X-100 in 0.1% sodium citrate for 2 min at 4°C. TdT labeling was performed with the commercially available kit, according to the manufacturer’s protocol. The apoptotic cells were directly detectable by their red color with fluorescence microscopy (Eclipse E400, Nikon Inc., Melville, NY). The images were captured with a CCD camera and processed with a digital fluorescence microscope (VB-6000, Keyence, Osaka, Japan).

### Nuclear magnetic resonance (NMR)

All NMR experiments were performed with a JEOL ECA-600 NMR spectrometer with a 5 mm inverse triple-resonance ^1^H/^13^C/X probe, with a z-axis pulsed-field gradient for two-dimensional (2D) experiments or with a 5 mm tunable double-resonance probe for 1D experiments, operating at 600.17 MHz for ^1^H, 150.91 MHz for ^13^C, and 242.95 MHz for ^31^P at 298 K. The ^1^H chemical shift was referenced to the residual CD_2_HOD signal at 3.30 ppm. The ^13^C chemical shift was referenced to the solvent CD_3_OD signal at 49.0 ppm. The ^31^P chemical shift was referenced to an external reference of 85% H_3_PO_4_ at 0.0 ppm. The NMR spectra were measured and processed using a standard pulse sequence and the Delta version 5.0 software (JEOL, USA).

### Liquid chromatography and time-of-flight mass spectrometry (LC/TOF-MS)

The seventh lipid fraction (F7) was injected into the mass spectrometer with a high-pressure LC system (Acquity UPLC System; Waters, MA), with a BEH C_18_ column (2.1 × 50 mm, 1.7 mm) at a flow rate of 0.25 mL/min. Gradient elution was conducted with mobile phases A (0.1% formic acid in water) and B (acetonitrile). The gradient used commenced with 40% mobile phase B, isocratic for 0.7 min; increased to 90% B over 5.3 min; isocratic for 2.5 min; and then back to 40% B over 0.5 min. All mass spectrometric analyses were performed with the SYNAPT G2 HDMS platform (Waters, Manchester, UK). A voltage (3.0 kV) was applied to the stainless steel electrospray ionization (ESI) capillary under positive ion conditions. The TOF analyzer was set to resolution mode with a resolving power of 20,000 at *m/z* 556 (leucine enkephalin) and the *m/z* range of 50–1500 was calibrated with sodium formate. The capillary, extraction cone, and cone voltages were set to 3 kV, 104 kV, and 4 kV, respectively. The desolvation gas (nitrogen) was used at a flow rate of 800 L/h, and the source and desolvation temperatures were set to 100°C and 250°C, respectively.

### Ozonolysis

Excess ozone was passed through a solution of the F7 lipid fraction (1.6 mg) in CD_3_OD (1.1 mL) at –78°C for 1.1 h. After nitrogen gas was bubbled through the solution for 10 min to remove the excess ozone, dimethyl sulfide (0.5 mL) was added slowly with stirring at –78°C. The mixture was allowed to warm gradually to room temperature and stirring was continued overnight. An aliquot (0.2 mL) was analyzed with LC/TOF-MS, and the remaining solution was concentrated to a syrupy gum (1.0 mg) *in vacuo*.

### Oxygen radical absorbance capacity (ORAC) assay

The ORAC assay was performed in triplicate on standard lipids and the extracts, according to the manufacturer’s recommendations (OxiSelect ORAC Activity Assay Kit; Cell Biolabs, San Diego, CA). The diluted samples (0, 2.5, 5, 10, 20, 30, 40, and 50 μM) were analyzed in triplicate in a 96-well plate. To each well were added 150 μL of fluorescein solution (0.036 mg/L; Cell Biolabs) and 25 μL of the diluted sample or 25 μL of the Trolox standard (6-hydroxy-2,5,7,8-tetramethylcroman-2-carboxylic acid; Cell Biolabs), and the plate was incubated for 30 min at 37°C, after which 25 μL of 2,2′-azobis-2-aminopropane dihydrochloride (AAPH) solution (80 mg/mL; Cell Biolabs) was added. The ORAC assay quantifies the inhibition (expressed as a percentage over time) of the fluorescence produced by peroxyl radicals generated at a constant rate by the thermal decomposition of AAPH. The antioxidant capacity is expressed in μmol Trolox equivalents (TE)/g, and is calculated from a Trolox standard curve.

### Statistical analysis

All statistical analyses were performed with the GraphPad Prism 5 software (GraphPad Software Inc., La Jolla, CA). The results are presented as means ± standard deviations. The significance of differences was evaluated with one-way analysis of variance (ANOVA) followed by Tukey’s multiple comparisons procedure or Student’s *t* test.

## Results

### Lipid fraction extracted with chloroform–water (4:1, v/v) prevented H_2_O_2_-induced cell death

**Fig 1:**
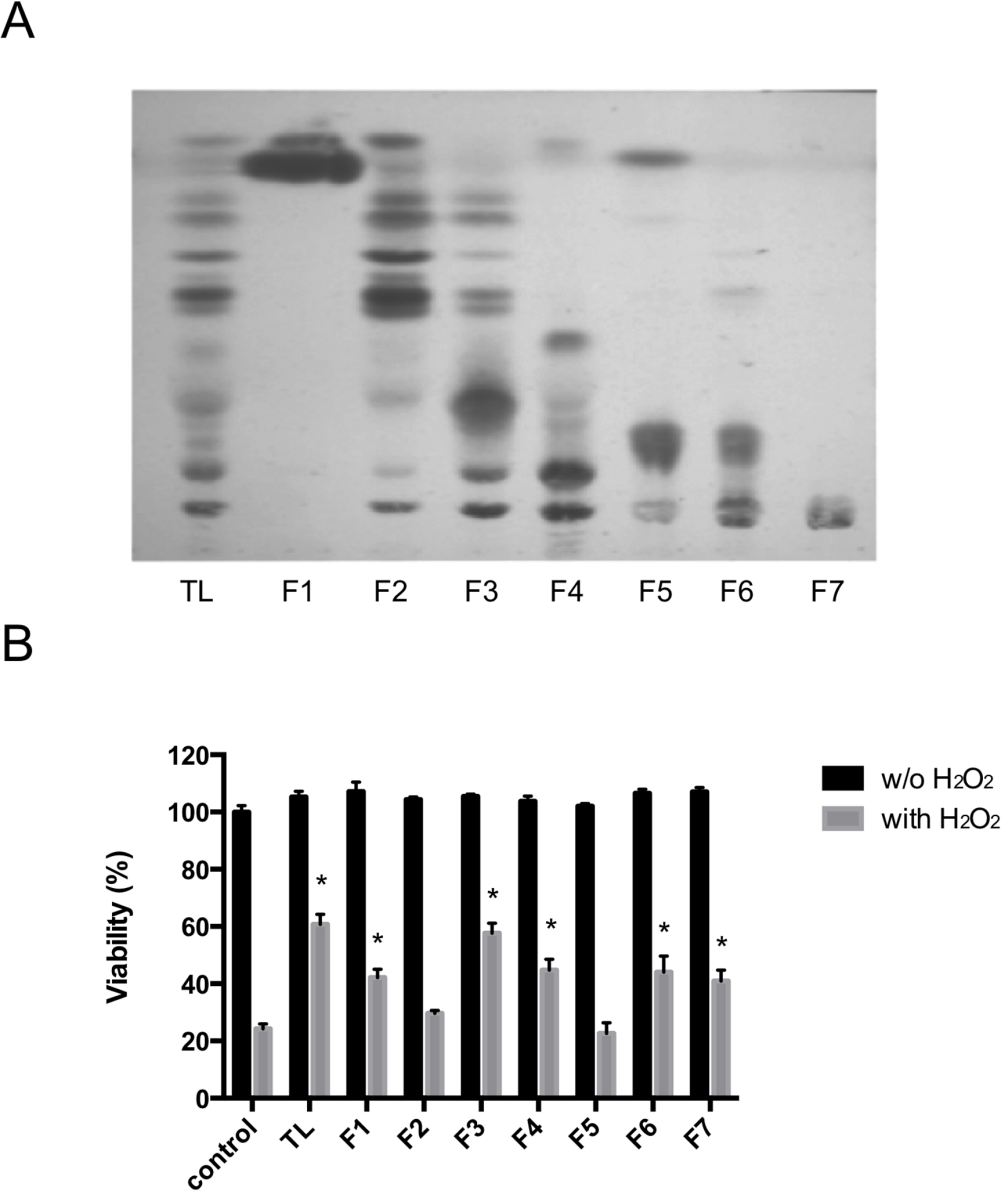
Thin-layer chromatogram (TLC) and cytoprotective effects of the lipids extracted from *Holothuria atra*. (A) TLC analysis of the lipids extracted from *H*. *atra* using a chloroform–methanol–water mixture (65:25:4, v/v/v). The total lipid (TL) was applied to a silica gel column in chloroform and eluted with: (F1) chloroform–methanol (4:1 v/v); (F2) chloroform–methanol (2:1 v/v); (F3) chloroform–methanol (1:1 v/v); (F4) chloroform–methanol (1:4 v/v); (F5) methanol; (F6) chloroform–methanol–water (3:6:1 v/v); or (F7) chloroform–water (4:1 v/v). (B) Viability of J774A.1 cells treated with the extracted lipids. J774A.1 cells were preincubated with each lipid fraction (10 μg/mL) for 12 h. H_2_O_2_ was then added to the cells (112.5 μM). After 12 h, cell viability was measured and expressed as the mean viability ± standard deviation of triplicate experiments. (*) According to Student’s *t* test, the difference between the lipid-treated cells and the untreated cells (control) was significant (*P* < 0.05).

**Fig 2:**
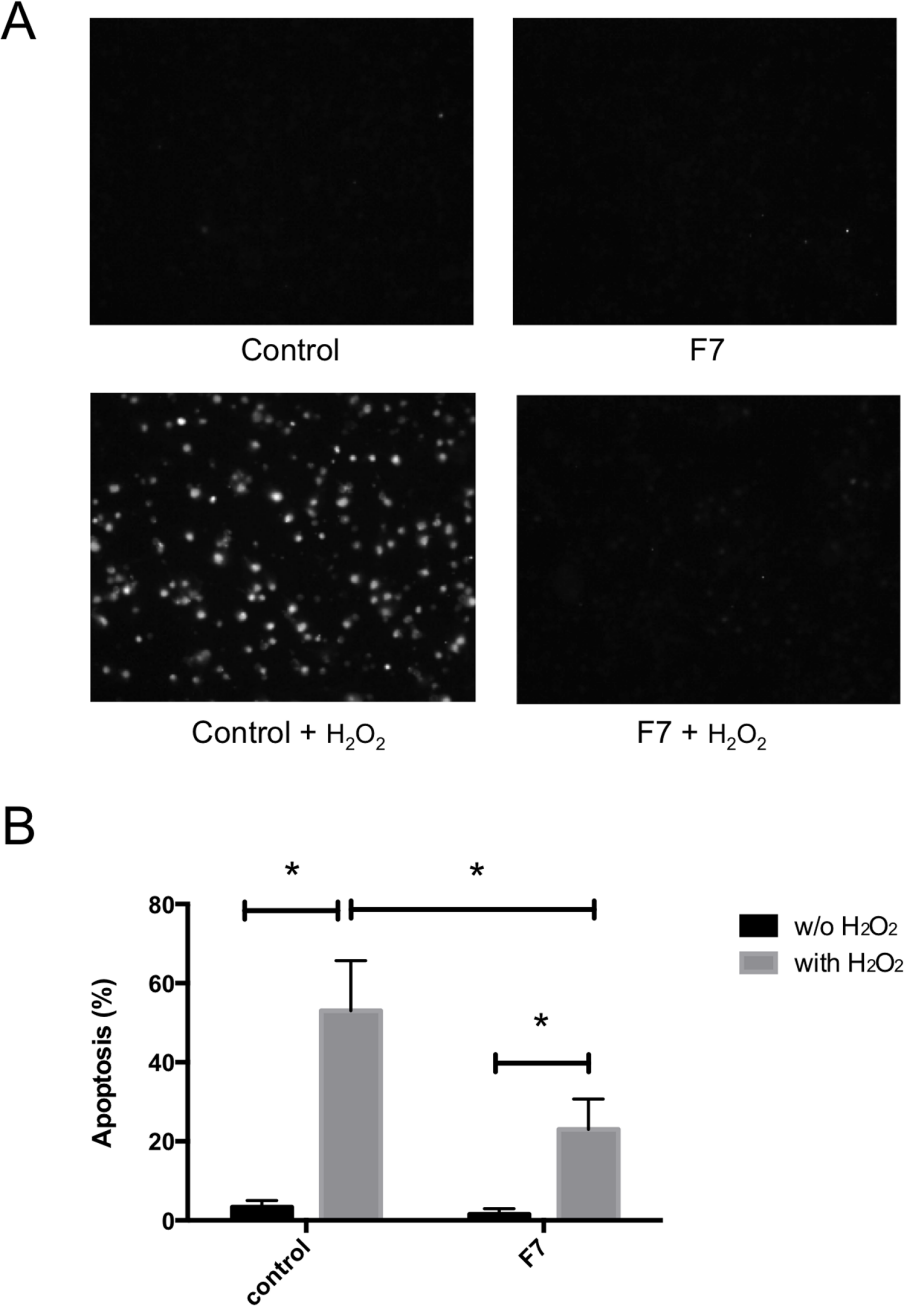
Antiapoptotic activity of the F7 fraction in H_2_O_2_-treated cells. J774A.1 cells were preincubated with the F7 lipid fraction for 12 h. H_2_O_2_ was then added to the cells (112.5 μM). After 12 h, the TUNEL-positive cells were quantified and the results expressed as the mean ± standard deviation of triplicate experiments. (A) TUNEL staining of J774A.1 cells treated with the F7 lipid fraction. (B) Frequency of apoptosis. (*) According to Student’s *t* test, the difference between the lipid-treated cells and untreated cells (control) was significant (*P* < 0.05).

Fig 1A shows the seven lipid fractions (F1–F7) eluted sequentially from a silica gel column with the following eluents: chloroform–methanol (4:1, 2:1, 1:1, 1:4, v/v), methanol, chloroform–methanol–water (3:6:1, v/v), and chloroform–water (4:1, v/v), respectively. We tested the protective activities of these lipid fractions in J774A.1 cells treated with H_2_O_2_ (Fig 1B). Pretreatment of the cells with TL from *H*. *atra* inhibited H_2_O_2_-induced cell death compared with that in the control cells treated with H_2_O_2_ only. The F1, F3, F4, F6, and F7 lipid fractions significantly protected the J774A.1 cells from H_2_O_2_ stress. Because F7 appeared as a single spot on TLC, we characterized this fraction in a further experiment. As shown in Fig 2, H_2_O_2_ treatment induced apoptosis in the J774A.1 cells of the control group. However, treatment with the F7 lipid fraction inhibited the apoptosis induced by H_2_O_2_. These results suggest that the F7 lipid fraction had antiapoptotic activity.

### Characterization of the lipid fraction extracted with chloroform–water (4:1, v/v)

**Fig 3:**
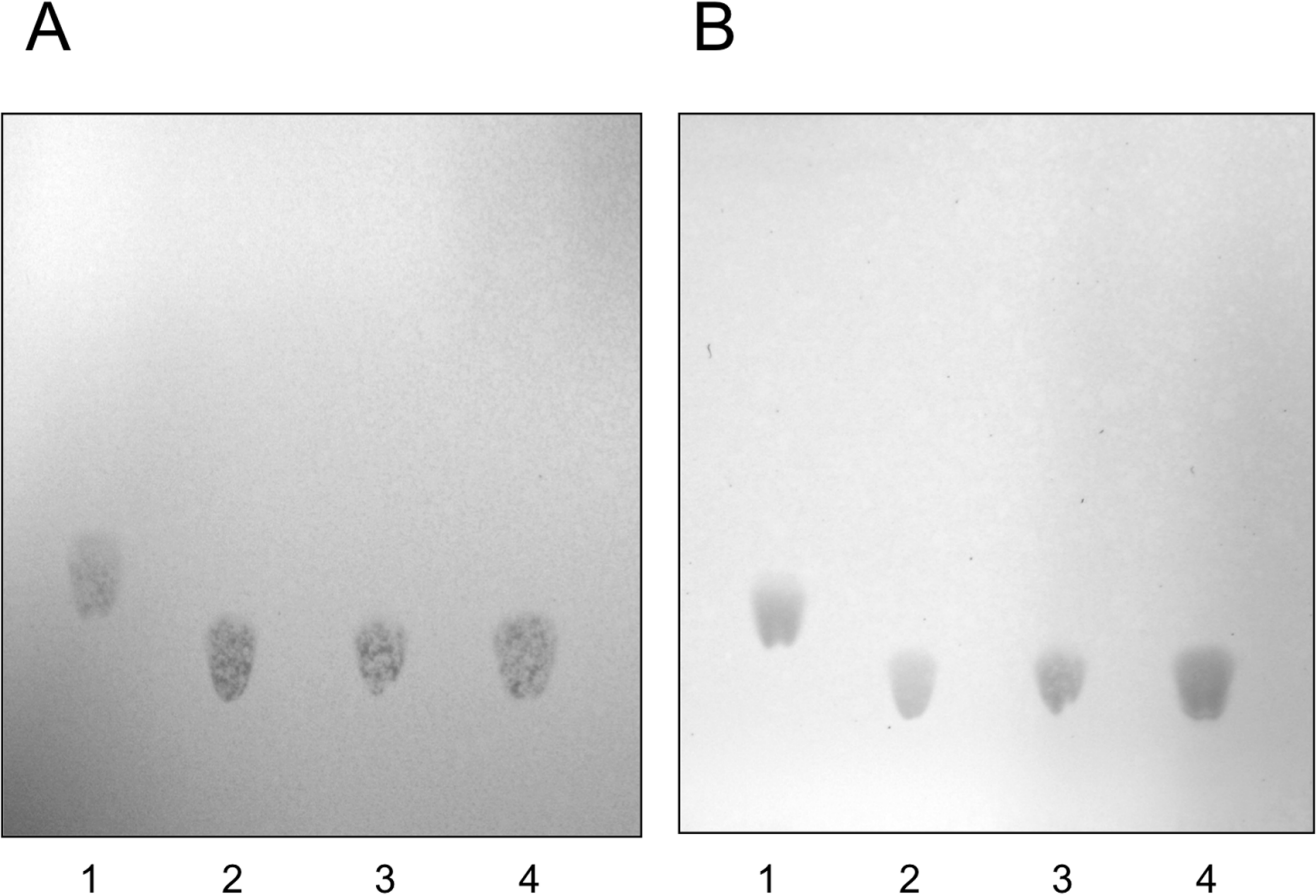
Thin-layer chromatogram of the F7 lipid fraction. The F7 lipid fraction was analyzed with Dittmer reagent (A) or 50% sulfuric acid (B) with a chloroform–methanol–water mixture (65:35:8, v/v/v). 1, Sphingomyelin; 2, Lyso-PAF; 3, F7 lipid fraction; 4, LPC.

To examine the contents of the F7 fraction, TLC using anthrone–sulfuric acid reagent and Dittmer reagent was performed to detect the carbohydrates and phospholipids, respectively, and 50% sulfuric acid was used to detect organic compounds. The anthrone–sulfuric acid reagent did not detect the spot of the F7 fraction or that of sphingomyelin, Lyso-PAF, or LPC. However, the spot of the F7 fraction was detected with Dittmer reagent, with Rf values similar to those for LPC and Lyso-PAF, but not with an Rf value similar to that of sphingomyelin (Fig 3), indicating the presence of phospholipids in the F7 fraction.

To analyze the phospholipids in the F7 fraction, we performed detailed NMR experiments, including 2D NMR with DQF-COSY, TOCSY, NOESY, HSQC, HSQC-TOCSY, ^1^H-^13^C HMBC, and ^1^H-^31^P HMBC (S1 File) [12]. The ^1^H NMR spectrum of the F7 fraction was quite similar to that of authentic Lyso-PAF (9*Z*)-C18:1. However, the integration values for the olefinic methine and allylic methylene signals were only half those expected for Lyso-PAF C18:1, and there were small minor signals. This indicates that the F7 fraction was a mixture of saturated and unsaturated Lyso-PAF, with minor amounts of other lipids. The connectivity between the glycerol unit and the alkyl chain portion through the ether bond in the Lyso-PAF structure was determined from long-range correlations in the ^1^H-^13^C HMBC spectra. The presence of a choline unit and the connectivity between the choline and glycerol units through phosphate were confirmed with NOESY, ^1^H-^13^C HMBC, and ^1^H-^31^P HMBC spectral data. However, it was difficult to clarify the double bond position with TOCSY, HSQC-TOCSY, and HMBC. A direct comparison showed that the ^13^C NMR spectrum of the F7 fraction was not identical to that of Lyso-PAF (9*Z*)-C18:1, whereas it closely matched the ^13^C signals from the C7–C12 portion of cis-5-dodecenoic acid, used as a model of the (11*Z*)-C18:1 alkyl chain. These observations suggested that the F7 fraction contained an isomer of Lyso-PAF C18:1, and its double bond position was tentatively assigned to C11. The ^13^C chemical shift values for the allylic methylene carbon signals at 28.1 ppm indicated that the stereochemistry of the double bond was *Z*. An LC/TOF-MS analysis of the F7 fraction (Table 1 and Supporting Information) showed two major peaks at retention times (RTs) of 4.95 and 5.83 min in the base peak ion chromatogram. The RT and *m/z* were identical to those of a standard sample, indicating that the peak at RT = 5.83 corresponded to Lyso-PAF C18:0 (**1**). The other main peak at RT = 4.95 was probably an isomer of Lyso-PAF (9*Z*)-C18:1 because it had the same *m/z* 530 for [M+Na]^+^, with a slightly shorter RT. Ozonolysis was performed to determine the position of the double bond in Lyso-PAF C18:1 (RT = 4.95). To clearly identify the expected dimethyl acetal derivative, the reaction was performed in CD_3_OD solution. The three products were identified with LC/TOF-MS as C11-carboxylic acid (**7**), C11-aldehyde (**8**), and its *d6*-dimethyl acetal derivative (**9**). These data are consistent with the NMR data described above. Therefore, the peak at RT = 4.95 was identified as Lyso-PAF (11*Z*)-C18:1 [13,14].

**Table 1:** LC/TOF-MS data for the F7 fraction and its ozonolysis products.

F7 fraction	RT (min)	Formula	Observed *m/z*	Theoretical [M+H]^+^ *m/z*	Observed *m/z*	Theoretical [M+Na]^+^ *m/z*
LPC C16:0 (**4**)	4.49	C_24_H_50_NO_7_P	496.3417	496.3398	518.3230	518.3217
Lyso-PAF C16:0 (**5**)	4.76	C_24_H_52_NO_6_P	482.3615	482.2605	504.3437	504.3424
Lyso-PAF (11*Z*)-C18:1 (**2**)	4.95	C_26_H_54_NO_6_P	508.3752	508.3762	530.3581	530.3581
LPC C18:0 (**3**)	5.57	C_26_H_54_NO_7_P	524.3742	524.3711	546.3538	546.3530
Lyso-PAF C18:0 (**1**)	5.83	C_26_H_56_NO_6_P	510.3920	510.3918	532.3733	532.3737
Lyso-PAF 17'-Me-C18:0 (**6**)	6.23	C_27_H_58_NO_6_P	524.4068	524.4075	546.3897	546.3894
standard samples	RT (min)	formula	Observe *m/z*	Theoretical [M+H]^+^ *m/z*	Observed *m/z*	Theoretical [M+Na]^+^ *m/z*
LPC C16:0 (**4**)	4.49	C_24_H_50_NO_7_P	496.3395	496.3398	518.3223	518.3217
LPC (9*Z*)-C18:1	4.76	C_26_H_52_NO_7_P	522.3547	522.3554	544.3392	544.3374
Lyso-PAF (9Z)-C18:1	5.00	C_26_H_54_NO_6_P	508.3744	508.3762	530.3583	530.3581
LPC C18:0 (**3**)	5.55	C_26_H_54_NO_7_P	524.3706	524.3711	546.3537	546.3530
Lyso-PAF C18:0 (**1**)	5.85	C_26_H_56_NO_6_P	510.3920	510.3918	532.3735	532.3737
ozonolysis products of F7 fraction	RT (min)	formula	Observe *m/z*	Theoretical [M+H]^+^ *m/z*	Observed *m/z*	Theoretical [M+Na]^+^ *m/z*
C11-carboxylic acid (**7**)	0.70	C_19_H_40_NO_8_P	442.2568	442.2564	ND	
C11-aldehyde (**8**)	0.78	C_19_H_40_NO_7_P	426.2614	462.2615	ND	
C11-*d6*-dimethyl acetal (**9**)	1.01	C_21_H_40_D_6_NO_8_P	478.3390	478.3410	ND	

ND; not done.

**Fig 4:**
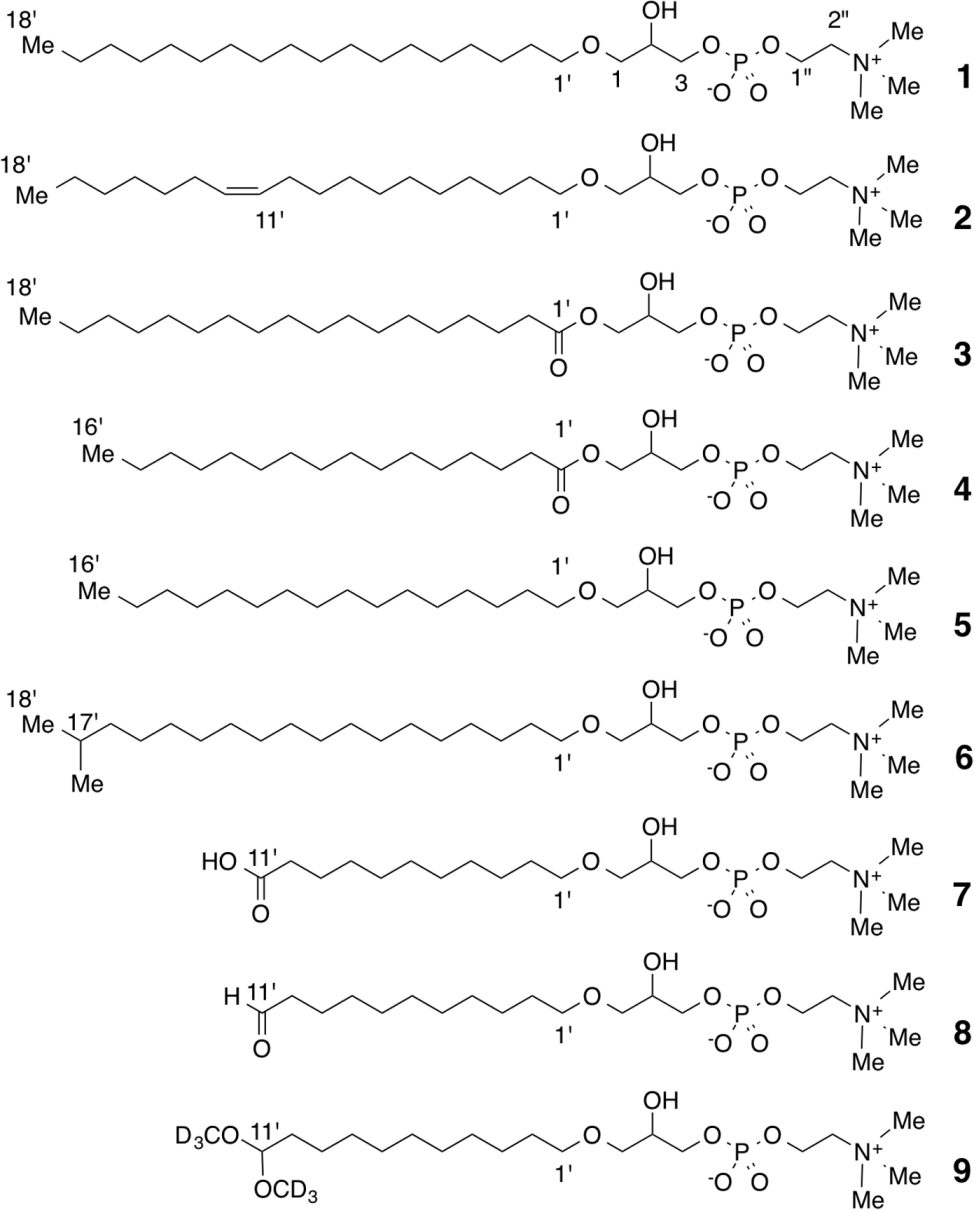
Structures of lysophospholipids. Compounds **1**–**6** were identified as Lyso-PAF and LPC in the F7 lipid fraction. Compounds **7**–**9** were the products of ozonolysis.

Some of the minor components were inferred to be LPC based on ^1^H-^1^H correlations in the DQF-COSY and TOCSY analyses of the glycerol and fatty acid units and the ^13^C NMR data assigned from the ^1^H-^13^C HSQC and HMBC spectra. LC/TOF-MS also detected LPC C18:0 (**3**), LPC C16:0 (**4**), Lyso-PAF C16:0 (**5**), and Lyso-PAF C19:0, and accurately determined their masses (Table 1 and Supporting Information). An odd number of alkyl chains in the linear form is relatively rare, so the minor signals in the NMR spectra were analyzed carefully. In the ^1^H-^13^C HMBC spectra, a minor doublet methyl signal at 0.87 ppm showed ^1^H-^13^C long-range correlations with carbon signals at 40.24 (CH_2_), 29.15 (CH), and 23.04 (CH_3_), and the last carbon was identical to the methyl carbon itself. These data, and especially the ^13^C NMR chemical shift values, suggested the presence of an isopropyl group at the end of the alkyl chain. Based on this evidence, the peak at RT = 6.23, Lyso-PAF C19:0, was tentatively assigned as the 17′-methyl derivative of Lyso-PAF C18:0 (**6**). In summary, NMR and LC/TOF-MS analyses identified Lyso-PAF C18:0 (**1**), Lyso-PAF (11*Z*)-C18:1 (**2**), LPC C18:0 (**3**), LPC C16:0 (**4**), Lyso-PAF C16:0 (**5**), and 17′-methyl Lyso-PAF C18:0 (**6**) in the F7 fraction (Fig 4). Based on the integration values of the ^1^H-NMR spectrum of the F7 fraction, the ratio of Lyso-PAF (compounds **1**, **2**, **5**, and **6**) to LPC (compounds **3** and **4**) was 88:12. The content of the minor compound **6** in the F7 fraction was estimated to be ca. 5% from the integration value for the doublet methyl signal. Determining the ratio of C18:0 to C16:0, such as (**1**):(**5**) and (**3**):(**4**), was impossible with NMR. However, the ratio of (**3**) or (**4**) was roughly estimated to be ca. 1:1 with a gas chromatographic analysis of the alkaline hydrolysate.

### Roles of Lyso-PAF and LPC in the cytoprotective and antioxidative activities of F7

**Fig 5:**
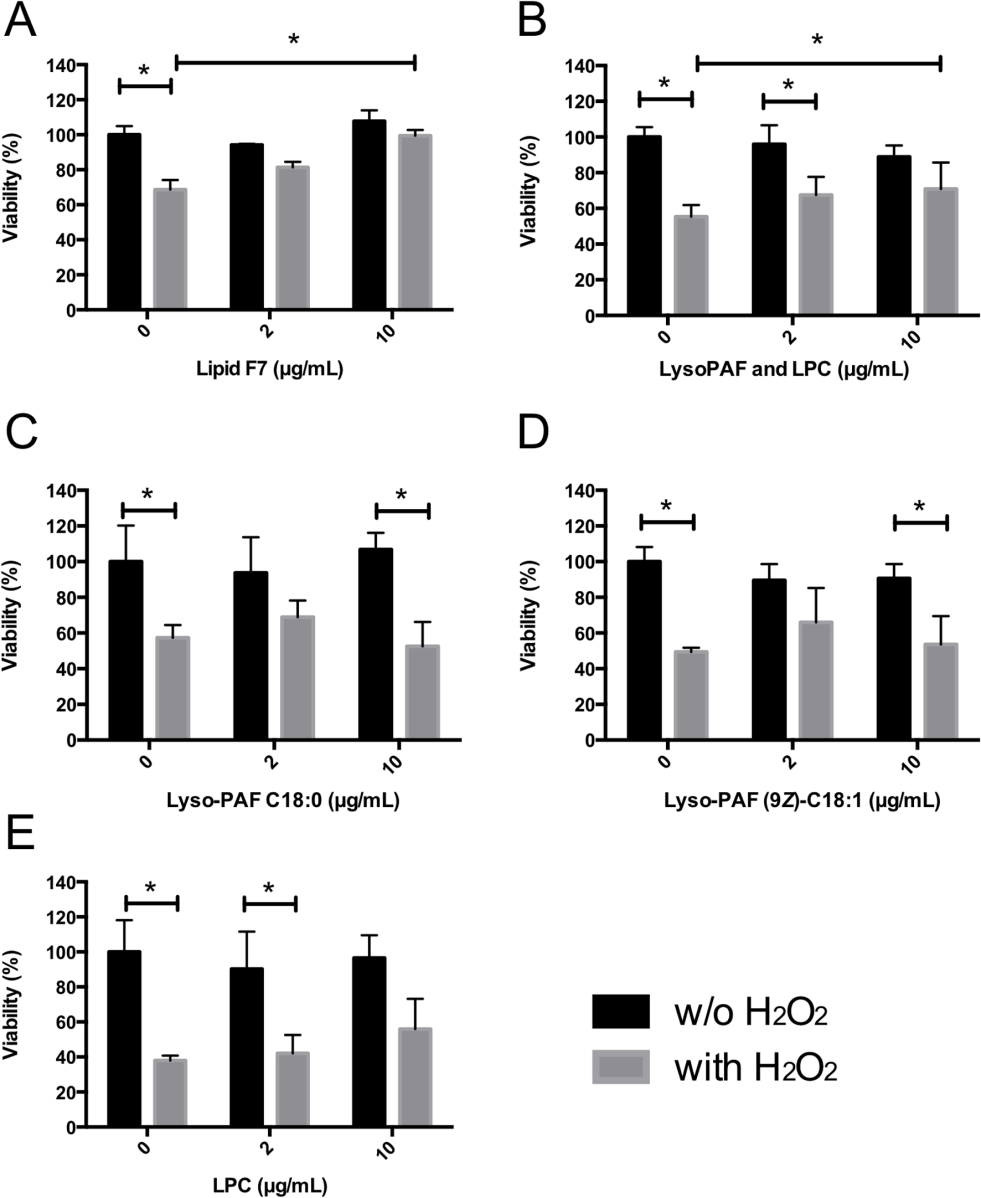
Viability of J774A.1 cells treated with LPC or Lyso-PAF. J774A.1 cells were preincubated with LPC or Lyso-PAF for 12 h, and H_2_O_2_ was then added to the cells (25 μM). After 12 h, cell viability was measured and is expressed as the mean viability ± standard deviation of triplicate experiments. (A) F7 lipid fraction. (B) Lipid mixture containing Lyso-PAF C18:0 (44%), Lyso-PAF (9*Z*)-C18:1 (44%), and LPC C18:0 (12%). (C) Lyso-PAF C18:0. (D) Lyso-PAF (9*Z*)-C18:1. (E) LPC. (*) Significant (*P* < 0.05) according to ANOVA followed by Tukey’s multiple comparisons.

**Fig 6:**
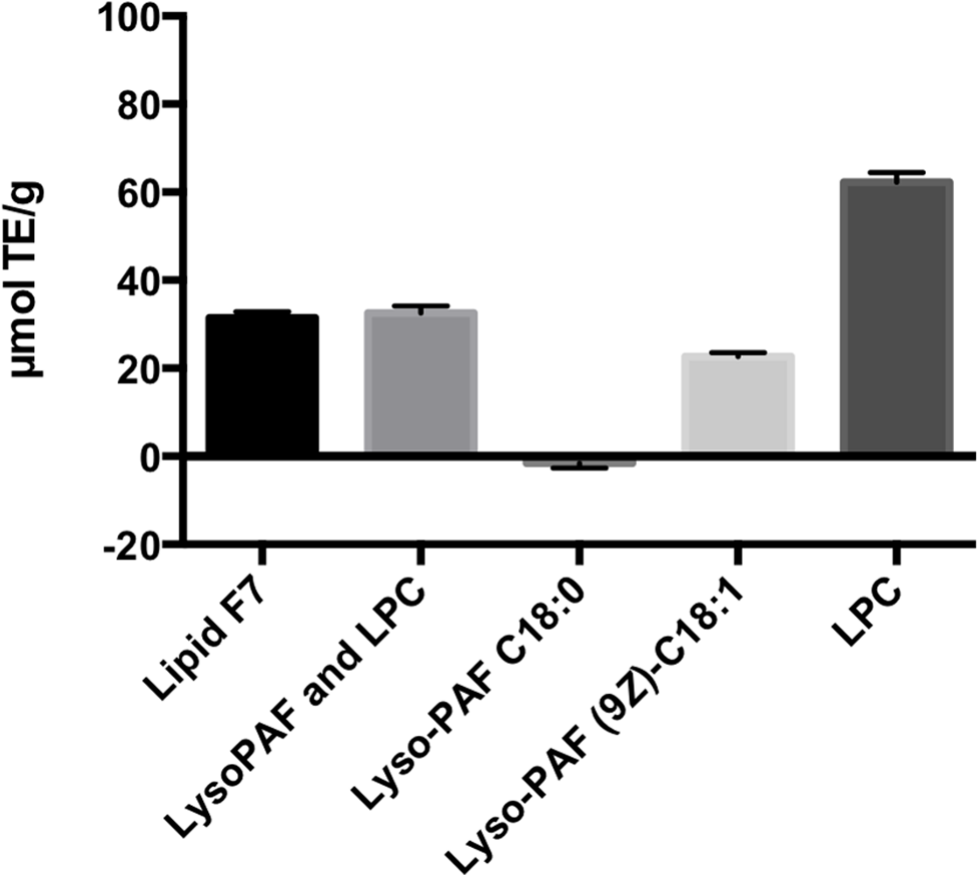
ORAC assay. Antioxidant capacities of the F7 lipid fraction and a lipid mixture containing Lyso-PAF C18:0 (44%), Lyso-PAF (9*Z*)-C18:1 (44%), and LPC C18:0 (12%). Lyso-PAF C18:0, Lyso-PAF (9*Z*)-C18:1, and LPC are expressed in μmol Trolox equivalents (TE)/g, calculated from a Trolox standard curve.

Commercially available C18 lipids were tested to confirm the roles of Lyso-PAF and LPC in the cytoprotection of J774A.1 cells (Fig 5). Treatment of the J774A.1 cells with the F7 fraction or with a 10 μg/mL lipid mixture containing Lyso-PAF C18:0 (44%), Lyso-PAF (9*Z*)-C18:1 (44%), and LPC C18:0 (12%) significantly increased cell viability in the presence of H_2_O_2_ compared with that of cells not treated with the lipids. However, treatment with Lyso-PAF C18:0, Lyso-PAF (9*Z*)-C18:1, or LPC C18:0 alone exerted no cytoprotective effect against H_2_O_2_ stress. In contrast, the ORAC assay revealed that Lyso-PAF (9*Z*)-C18:1 and LPC C18:0 each had antioxidative activity (Fig 6). These data indicate that the additive effects of Lyso-PAF and LPC may be required for their cytoprotective effect against oxidative stress.

## Discussion

Various species of invertebrates contain large amounts of both phosphocholine and phosphoethanolamine. Platelet-activating factor (PAF)-like lipids are also widely distributed among various invertebrates. Therefore, phospholipids, including PAF, may be physiologically important molecules, particularly for invertebrates [15]. In this study, we detected Lyso-PAF and LPC in the lipid fraction with antioxidative activity extracted from the sea cucumber *H*. *atra* for the first time. NMR and LC/TOF-MS analyses identified Lyso-PAF C18:0 (**1**), Lyso-PAF (11*Z*)-C18:1 (**2**), LPC C18:0 (**3**), LPC C16:0 (**4**), Lyso-PAF C16:0 (**5**), and 17′-methyl Lyso-PAF C18:0 (**6**) in the bioactive fraction. A Lyso-PAF-related molecule has also been detected in the sea cucumber *Cucumaria frondosa* [16]. Interestingly, a series of Lyso-PAF analogues and LPCs were isolated from the marine sponge *Spirastrella abata* [14]. LPC C16:0 and Lyso-PAF (11*Z*)-C18:1 have also been identified in *S*. *abata* [14] and an Australian sponge, *Crella incrustans* [17]. These results suggest that Lyso-PAF analogues and LPCs are widely distributed among marine invertebrates, and some compounds may be species-specific.

Lyso-PAF is produced when PAF is cleaved at the *sn*-2 position by phospholipase A2. A highly specific PAF-acetyltransferase then adds an acetyl group to the *sn*-2 position to yield PAF [18]. The presence of acetyltransferase activity has been confirmed in several marine invertebrates [15], suggesting the biosynthesis of Lyso-PAF in the lower animals. Importantly, lyso-PAF can act as a bioactive lipid, with functions opposite those of PAF [19]. The PAF generated by basophils induces platelet aggregation and anaphylaxis [20,21], and the PAF produced by neutrophils plays important roles in inflammation, including in the activation of NADPH oxidase [19]. The accumulation of PAF is also thought to be a principal initiator of neuronal dysfunction and death in human immunodeficiency virus (HIV)-associated dementia and a secondary mediator of neural loss in ischemia and epilepsy [22,23]. Therefore, the intrinsic regulatory mechanisms that balance PAF activities are potential targets for therapeutic interventions. Lyso-PAF also seems to have antioxidative activity. In this study, treatment with Lyso-PAF C18:0 or Lyso-PAF (9*Z*)-C18:1 alone exerted no cytoprotective effect against H_2_O_2_ stress on J774A.1 cells. In contrast, according to an ORAC assay, Lyso-PAF (9*Z*)-C18:1 has antioxidative activity. Further research is required to evaluate the different activities of saturated and unsaturated Lyso-PAF.

In mammals, LPC is a highly abundant bioactive lysoglycerophospholipid in the circulation, predominantly associated with albumin [24] and lipoproteins, such as oxidized low-density lipoprotein (oxLDL) [25]. The hydrolysis of the fatty acid of membrane phosphatidylcholine at the sn-2 position by the superfamily of phospholipase A2 enzymes generates LPC and fatty acids [26]. Although the activity of phospholipase A2 has not been demonstrated in the sea cucumber, the expression of phospholipase has been confirmed with a proteomic analysis in the sea cucumber *Apostichopus japonicus* coelomocytes after *Vibrio splendidus* infection [27]. This suggests the presence of the biosynthetic pathway for LPC in the sea cucumber *H*. *atra*. Generally, LPC is an important signaling lipid with diverse functions in inflammation. LPC is considered a causative factor in atherosclerosis, the promotion of demyelination in the nervous system, the induction of hepatitis, and protection from sepsis. LPC also acts as a chemoattractant signal for phagocytic cells, which function as immunoregulatory cells [28]. Our results indicate that the treatment of J774A.1 cells with LPC C18:0 conferred no cytoprotective effect against H_2_O_2_ stress, whereas the ORAC assay demonstrated the antioxidative activity of LPC C18:0. Although stearoyl LPC significantly attenuates circulating high-mobility group box 1 (HMGB1) levels in endotoxemia and sepsis by suppressing the release of endotoxin-induced HMGB1 from macrophages/monocytes [29], there has been no report of the antioxidative activity of LPC. Therefore, the mechanism underlying the antioxidative activity of LPC must be addressed in a future study.

Although the effects of the lysophospholipids of sea cucumbers are largely unknown, this study has shown that Lyso-PAF and LPC in the sea cucumber *H*. *atra* have cytoprotective activity *in vitro*. Importantly, the additive effects of Lyso-PAF and LPC are required for their cytoprotective activity against oxidative stress. Among the analogues of lysophospholipids, Lyso-PAF exhibits cancerostatic properties [30,31] and potent antimicrobial activity [32]. Our results suggest that the combination of Lyso-PAF and LPC from the sea cucumber has activities that can control inflammation in animals, including humans. Lyso-PAF and LPC analogues are abundant in invertebrates and are widespread among marine invertebrates [15], so our novel findings should prompt the investigation of the therapeutic uses of lysophospholipids from these organisms.

## Supporting Information

S1 File Contains Table A 1H and 13C NMR data for Compounds 1–4 and 6. Fig A 1H NMR spectra of F7 fraction, and authentic Lyso-PAF (9Z)-C18:1. Fig B 13C NMR spectra of F7 fraction, and authentic Lyso-PAF (9Z)-C18:1 and cis-5-dodecenoic acid. Fig C DQF-COSY spectrum of F7 fraction. Fig D TOCSY spectrum of F7 fraction. Fig E NOESY spectrum of F7 fraction. Fig F HSQC spectrum of F7 fraction. Fig G HSQC-TOCSY spectrum of F7 fraction. Fig H 1H-13C HMBC spectrum of F7 fraction. Fig I 1H-31P HMBC spectrum of F7 fraction. Fig J Base peak ion chromatograms and mass chromatograms on LC/TOF-MS. Fig K Mass spectrum for RT 5.83 of F7 fraction, Lyso-PAF C18:0 (1). Fig L Mass spectrum for RT 4.95 of F7 fraction, Lyso-PAF (11Z)-C18:1 (2). Fig M Mass spectrum for RT 5.57 of F7 fraction, LPC C18:0 (3) Fig N Mass spectrum for RT 4.49 of F7 fraction, LPC C16:0 (4) Fig O Mass spectrum for RT 4.76 of F7 fraction, Lyso-PAF C16:0 (5). Fig P Mass spectrum for RT 6.23 of F7 fraction, Lyso-PAF 17’- Methyl-C18:0 (6). Fig Q Mass spectrum for Lyso-PAF C18:0 (1) of a standard mixture. Fig R Mass spectrum for Lyso-PAF (9Z)-C18:1 of a standard mixture. Fig S Mass spectrum for LPC C18:0 (3) of a standard mixture. Fig T Mass spectrum for LPC C16:0 (4) of a standard mixture. Fig U Mass spectrum for LPC (9Z)-C18:1 of a standard mixture. Fig V Mass spectrum for RT 0.70 of ozonolysis products, C11-carboxylic acid (7). Fig W Mass spectrum for RT 0.78 of ozonolysis products, C11-aldehyde (8). Fig X Mass spectrum for RT 1.01 of ozonolysis products, C11-d6-dimethyl acetal (9).
(PDF)
