# Supplementary material for: Cytoprotective Effects of Lysophospholipids from Sea Cucumber Holothuria atra
Source: PLoS One. 2015 Aug 14;10(8):e0135701. doi: 10.1371/journal.pone.0135701 (PMC4537208; doi:10.1371/journal.pone.0135701)

List of contents

Table A ^{1}H and ^{13}C NMR data for Compounds **1**-**4** and **6**.
Fig. A ^{1}H NMR spectra of F7 fraction, and authentic Lyso-PAF (9Z)-C18:1.
Fig. B ^{13}C NMR spectra of F7 fraction, and authentic Lyso-PAF (9*Z*)-C18:1 and cis-5-dodecenoic acid.
Fig. C DQF-COSY spectrum of F7 fraction.
Fig. D TOCSY spectrum of F7 fraction.
Fig. E NOESY spectrum of F7 fraction.
Fig. F HSQC spectrum of F7 fraction.
Fig. G HSQC-TOCSY spectrum of F7 fraction.
Fig. H ^{1}H-^{13}C HMBC spectrum of F7 fraction.
Fig. I ^{1}H-^{31}P HMBC spectrum of F7 fraction.
Fig. J Base peak ion chromatograms and mass chromatograms on LC/TOF-MS.
Fig. K Mass spectrum for RT 5.83 of F7 fraction, Lyso-PAF C18:0 (**1**).
Fig. L Mass spectrum for RT 4.95 of F7 fraction, Lyso-PAF (11*Z*)-C18:1 (**2**).
Fig. M Mass spectrum for RT 5.57 of F7 fraction, LPC C18:0 (**3**)
Fig. N Mass spectrum for RT 4.49 of F7 fraction, LPC C16:0 (**4**)
Fig. O Mass spectrum for RT 4.76 of F7 fraction, Lyso-PAF C16:0 (**5**).
Fig. P Mass spectrum for RT 6.23 of F7 fraction, Lyso-PAF 17'-Methyl-C18:0 (**6**).
Fig. Q Mass spectrum for Lyso-PAF C18:0 (**1**) of a standard mixture.
Fig. R Mass spectrum for Lyso-PAF (9*Z*)-C18:1 of a standard mixture.

Fig. S Mass spectrum for LPC C18:0 (**3**) of a standard mixture.
Fig. T Mass spectrum for LPC C16:0 (**4**) of a standard mixture.
Fig. U Mass spectrum for LPC (9*Z*)-C18:1 of a standard mixture.
Fig. V Mass spectrum for RT 0.70 of ozonolysis products, C11-carboxylic acid (**7**).
Fig. W Mass spectrum for RT 0.78 of ozonolysis products, C11-aldehyde (**8**).
Fig. X Mass spectrum for RT 1.01 of ozonolysis products, C11-*d6*-dimethyl acetal (**9**).

Table A, ^{1}H and ^{13}C NMR data of compounds 1 – 4 and 6 (in CD_3OD). $

	**1**		**2**		**3**		**4**		**6**	
No	^{13}C	^{1}H	^{13}C	^{1}H	^{13}C	^{1}H	^{13}C	^{1}H	^{13}C	^{1}H
1	72.92	3.49 dd (9.6, 5.5)	72.92	3.49 dd (9.6, 5.5)	66.24	4.17 dd (11.5, 4.6)	66.24	4.17 dd (11.5, 4.6)	72.92	3.49 dd (9.6, 5.5)
		3.44 dd (9.6, 5.5)		3.44 dd (9.6, 5.5)		4.10 dd (11.5, 6.5)		4.10 dd (11.5, 6.5)		3.44 dd (9.6, 5.5)
2	71.03 d (7.5)	3.88 m	71.03 d (7.5)	3.88 m	69.84 d (9.0)	3.96 m	69.84 d (9.0)	3.96 m	71.03 d (7.5)	3.88 m
3	68.49 d (6.0)	3.93 ddd (10.3, 5.9, 3.6)	68.49 d (6.0)	3.93 ddd (10.3, 5.9, 3.6)	67.81 d (7.5)	3.89 m	67.81 d (7.5)	3.89 m	68.49 d (6.0)	3.93 ddd (10.3, 5.9, 3.6)
		3.84 ddd (10.3, 6.4, 5.5)		3.84 ddd (10.3, 6.4, 5.5)						3.84 ddd (10.3, 6.4, 5.5)
1'	72.69	3.45 t (7.3)	72.69	3.45 t (7.3)	175.38	----	175.38	----	72.69	3.45 t (7.3)
2'	30.66	1.56 m	30.66	1.56 m	34.92	2.34 t (7.6)	34.92	2.34 t (7.6)	30.66	1.56 m
3'	27.23	1.34 m	27.23	1.34 m	25.99	1.61 m	25.99	1.61 m	27.23	1.34 m
4' – 8'	30.77*	1.28 m	30.77*	1.28 m	31.09 – 30.23#	1.28 m	31.09 – 30.23#	1.28 m	31.09 – 30.23#	1.28 m
9'	30.77*	1.28 m	30.84*	1.33 m	30.77*	1.28 m	30.77*	1.28 m	31.09 – 30.23#	1.28 m
10'	30.77*	1.28 m	28.11**	2.02 m	30.77*	1.28 m	30.77*	1.28 m	31.09 – 30.23#	1.28 m
11'	30.77*	1.28 m	130.84	5.33 m	30.77*	1.28 m	30.77*	1.28 m	31.09 – 30.23#	1.28 m
12'	30.77*	1.28 m	130.84	5.33 m	30.77*	1.28 m	30.63*	1.28 m	31.09 – 30.23#	1.28 m
13'	30.77*	1.28 m	28.14**	2.02 m	30.77*	1.28 m	30.46*	1.28 m	31.09 – 30.23#	1.28 m
14'	30.63*	1.28 m	30.77*	1.33 m	30.63*	1.28 m	33.07	1.28 m	31.09 – 30.23#	1.28 m
15'	30.46*	1.28 m	30,03	1.28 m	30.46*	1.28 m	23.72	1.29 m	28.53	1.28 m
16'	33.07	1.28 m	32.93	1.28 m	33.07	1.28 m	14.44	0.89 t (7.2)	40.24	1.17 m
17'	23.72	1.29 m	23.72	1.29 m	23.72	1.29 m	___	___	29.15	1.52 m
18'	14.44	0.89 t (7.2)	14.44	0.90 t (7.2)	14.44	0.89 t (7.2)	___	___	23.04	0.87 d (6.9)
1"	60.37 d (4.5)	4.28 m	60.37 d (4.5)	4.28 m	60.42 d (4.5)	4.28 m	60.42 d (4.5)	4.28 m	60.37 d (4.5)	4.28 m
2"	67.50 brs	3.63 m	67.50 brs	3.63 m	67.50 brs	3.63 m	67.50 brs	3.63 m	67.50 brs	3.63 m
N-CH_3	54.68 t (3.8)	3.21 s	54.68 t (3.8)	3.21 s	54.68 t (3.8)	3.21 s	54.68 t (3.8)	3.21 s	54.68 t (3.8)	3.21 s

$ ^{1}H (600MHz), ^{13}C (150MHz). # separated small signals 31.09, 31.03, 30.59, 30.43, 30.23 *, ** Exchangeable.

Fig. A-1 ^{1}H NMR Spcetra

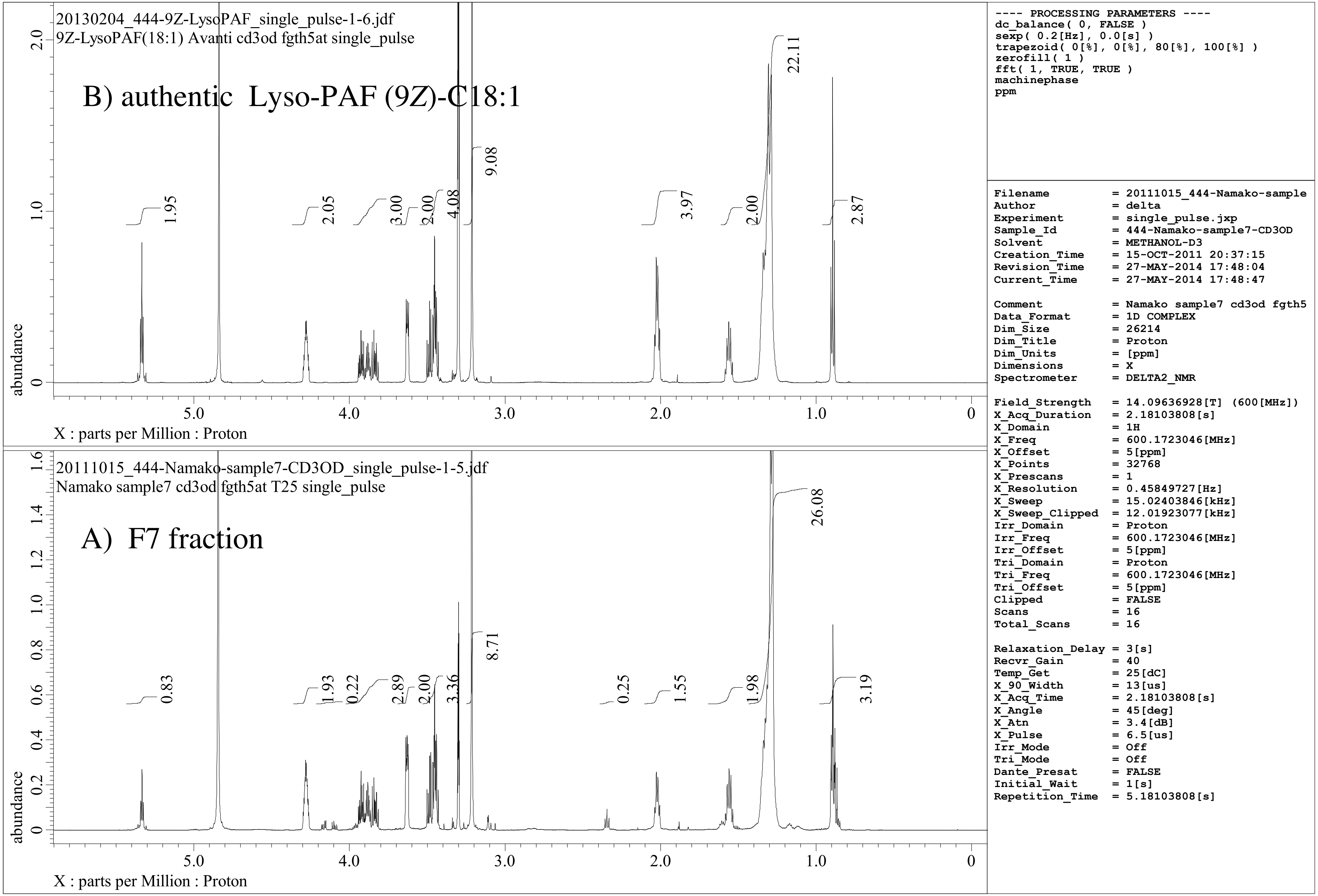

Fig. A-2 ^{1}H NMR Spectra (Expanded for regions of glycero and choline)

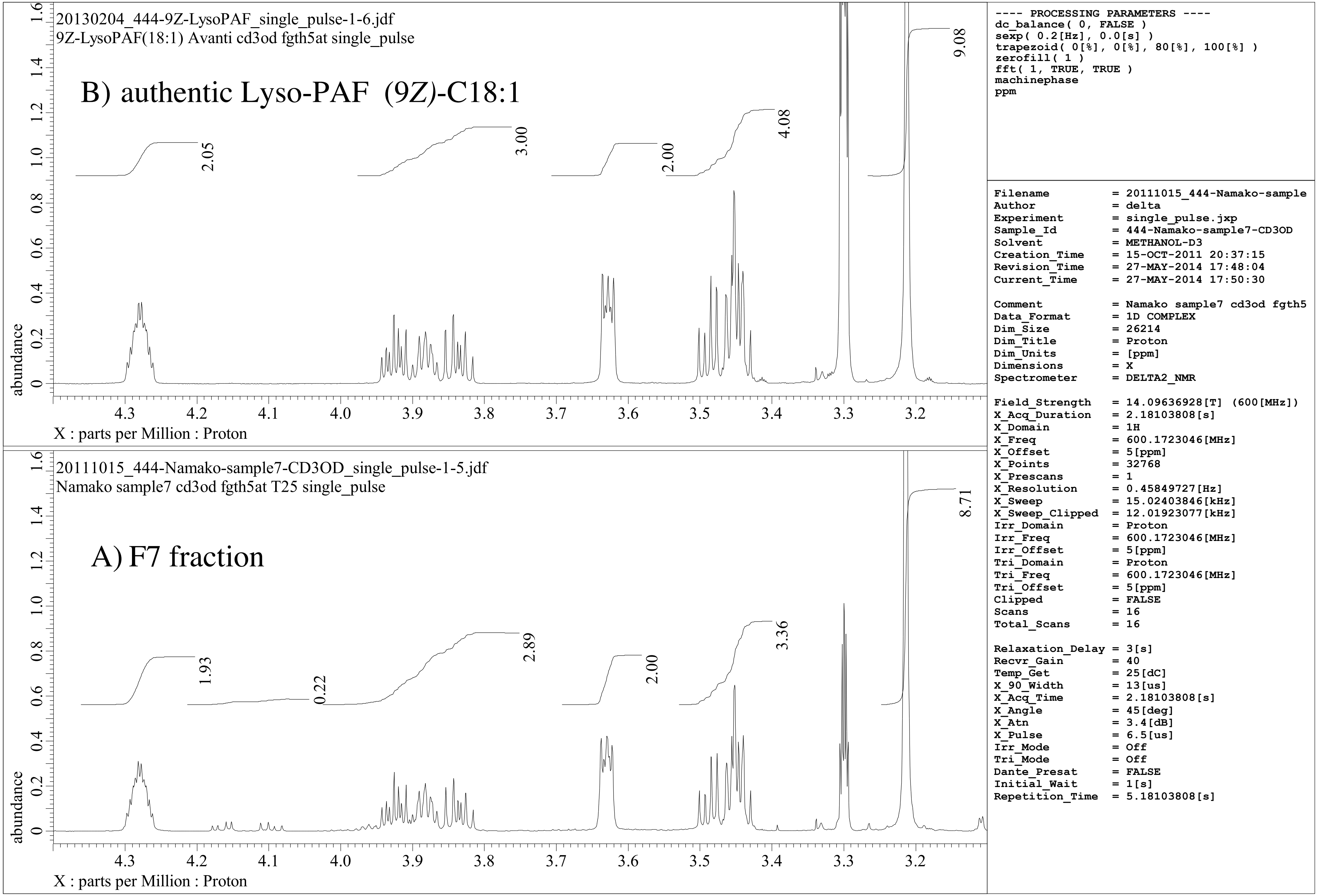

Fig. B-1 ^{13}C NMR Spectra

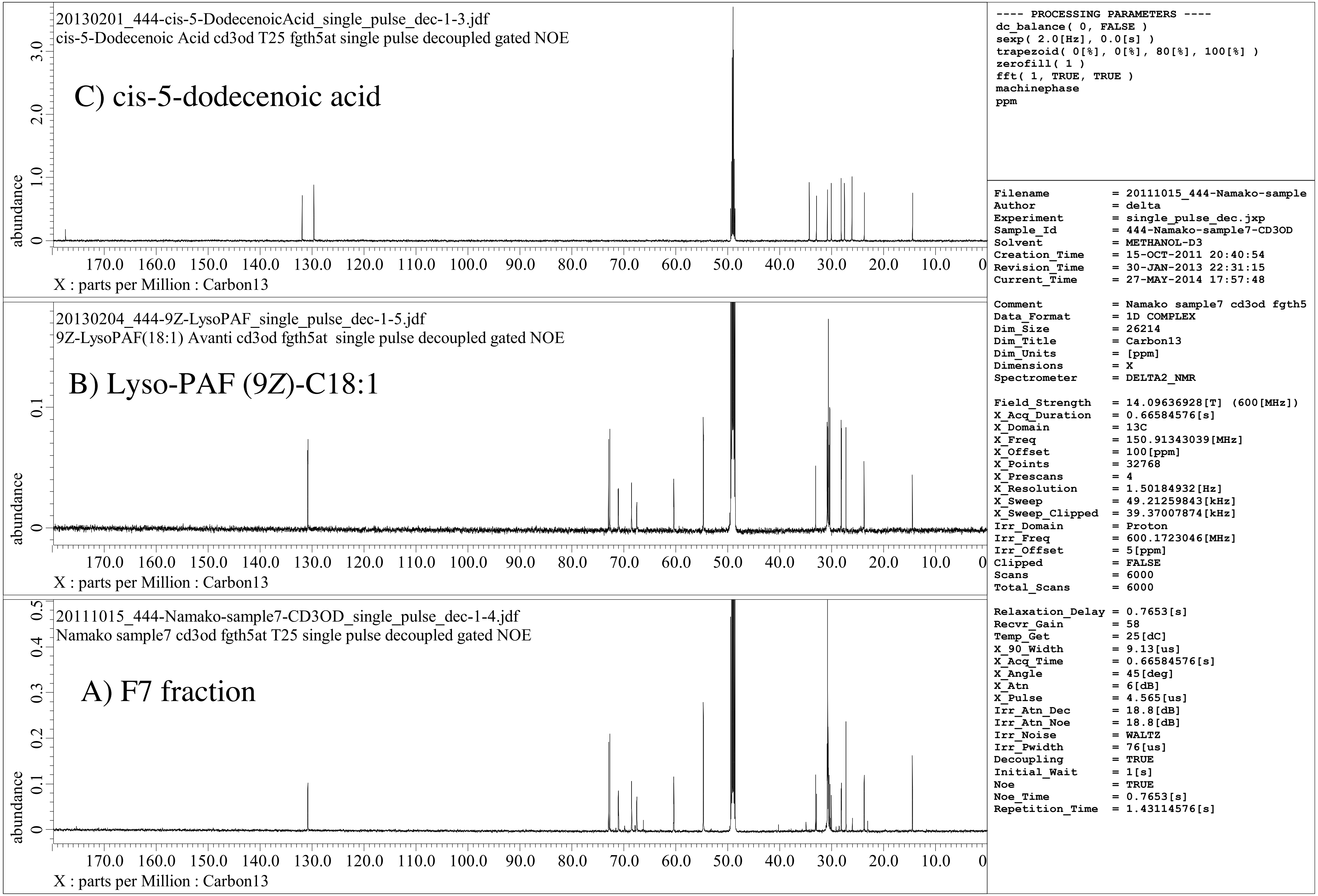

Fig. B-2 ^{13}C NMR Spectra (Expanded for upfield region)

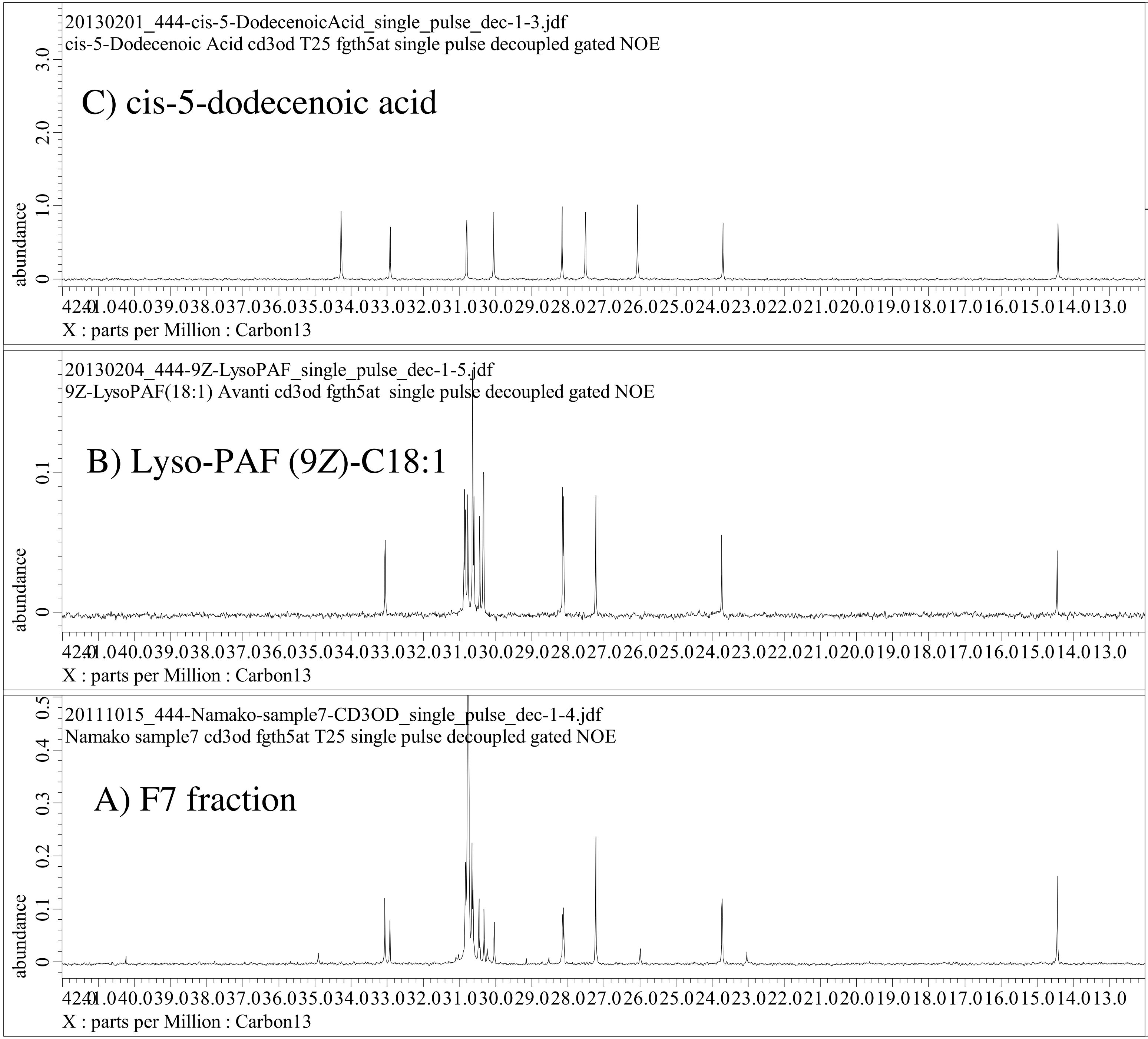


```
---- PROCESSING PARAMETERS ----
dc_balance( 0, FALSE )
sexp( 2.0[Hz], 0.0[s] )
trapezoid( 0[%], 0[%], 80[%], 100[%] )
zerofill( 1 )
fft( 1, TRUE, TRUE )
machinephase
ppm

Filename           = 20111015_444-Namako-sample
Author             = delta
Experiment         = single_pulse_dec.jxp
Sample_Id          = 444-Namako-sample7-CD3OD
Solvent            = METHANOL-D3
Creation_Time      = 15-OCT-2011 20:40:54
Revision_Time      = 30-JAN-2013 22:31:15
Current_Time       = 27-MAY-2014 17:58:41

Comment            = Namako sample7 cd3od fgth5
Data_Format        = 1D COMPLEX
Dim_Size           = 26214
Dim_Title          = Carbon13
Dim_Units          = [ppm]
Dimensions         = X
Spectrometer       = DELTA2_NMR

Field_Strength     = 14.09636928[T] (600[MHz])
X_Acq_Duration     = 0.66584576[s]
X_Domain           = 13C
X_Freq             = 150.91343039[MHz]
X_Offset           = 100[ppm]
X_Points           = 32768
X_Prescans         = 4
X_Resolution       = 1.50184932[Hz]
X_Sweep            = 49.21259843[kHz]
X_Sweep_Clipped    = 39.37007874[kHz]
Irr_Domain         = Proton
Irr_Freq           = 600.1723046[MHz]
Irr_Offset         = 5[ppm]
Clipped            = FALSE
Scans              = 6000
Total_Scans        = 6000

Relaxation_Delay   = 0.7653[s]
Recvr_Gain         = 58
Temp_Get           = 25[dC]
X_90_Width         = 9.13[us]
X_Acq_Time         = 0.66584576[s]
X_Angle            = 45[deg]
X_Atn              = 6[dB]
X_Pulse            = 4.565[us]
Irr_Atn_Dec        = 18.8[dB]
Irr_Atn_Noe        = 18.8[dB]
Irr_Noise          = WALTZ
Irr_Pwidth         = 76[us]
Decoupling         = TRUE
Initial_Wait       = 1[s]
Noe                = TRUE
Noe_Time           = 0.7653[s]
Repetition_Time    = 1.43114576[s]
```

Fig. C DQF-COSY spectrum of F7 fraction

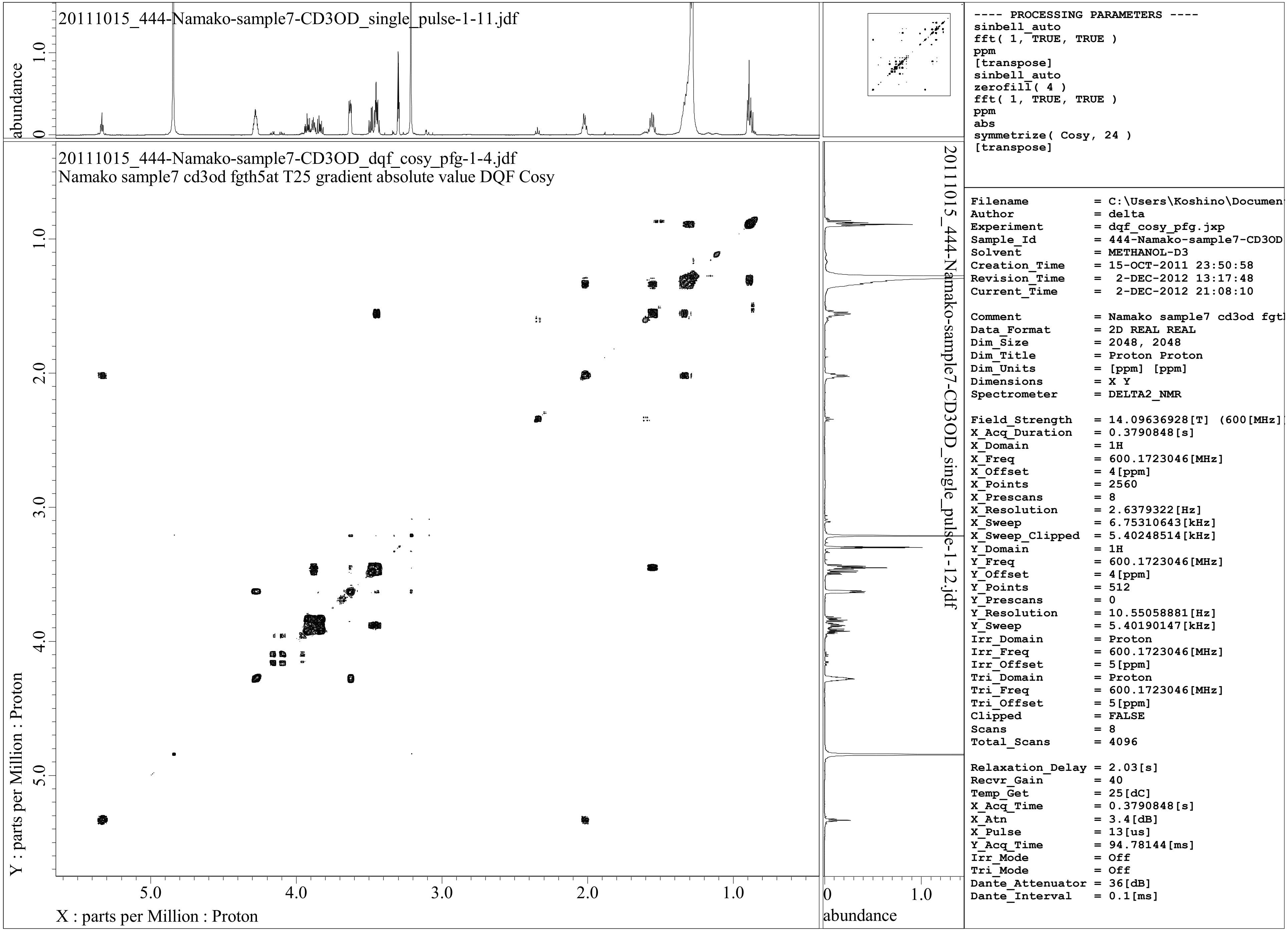

Fig. D TOCSY spectrum of F7 fraction

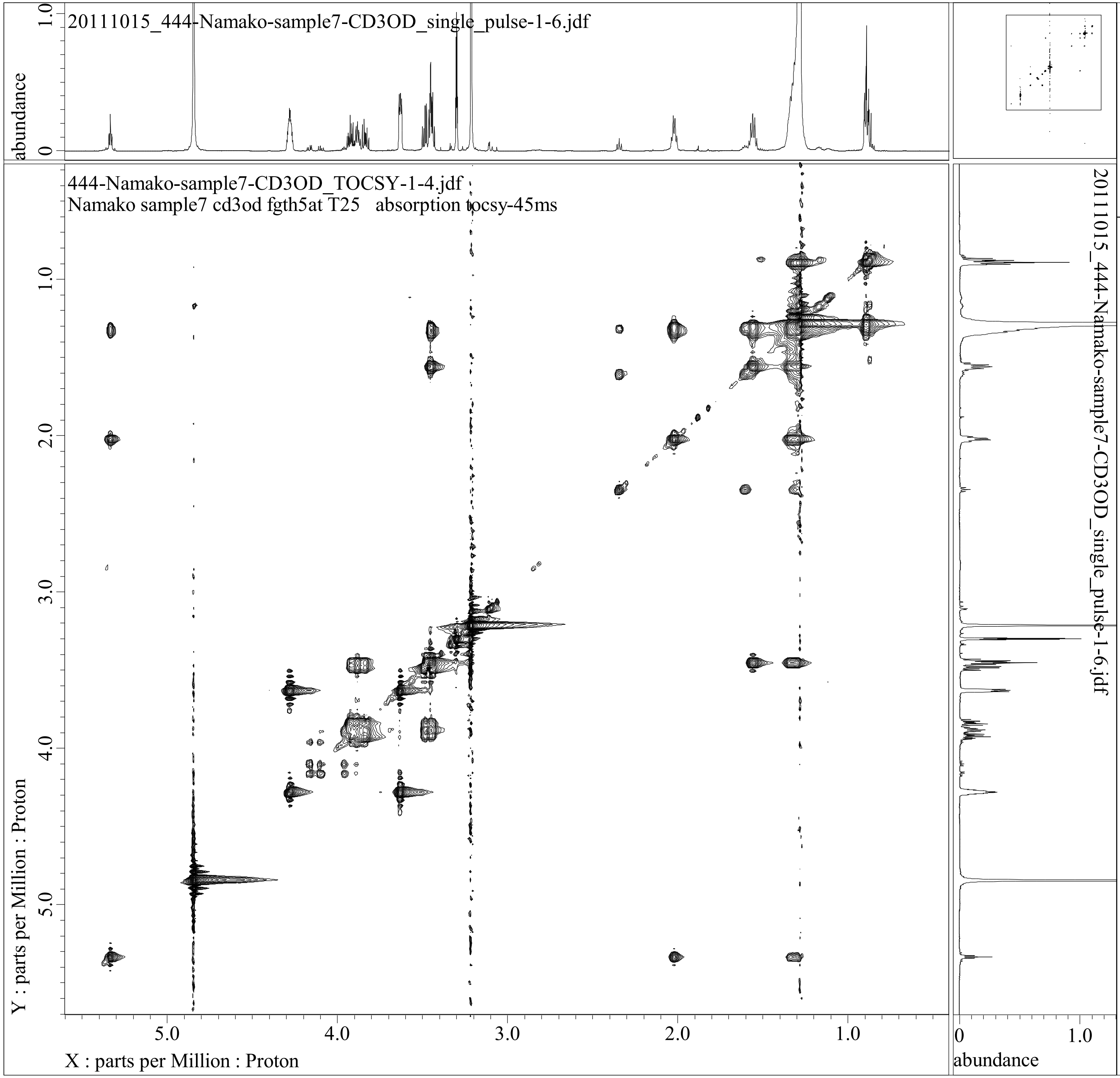


```
---- PROCESSING PARAMETERS ----
sexp( 5.0[Hz], 0.0[s] )
trapezoid( 0[%], 0[%], 80[%], 100[%] )
zerofill( 1 )
fft( 1, TRUE, TRUE )
ppm
machinephase
[transpose]
sexp( 5.0[Hz], 0.0[s] )
trapezoid( 0[%], 0[%], 80[%], 100[%] )
zerofill( 2 )
fft( 1, TRUE, TRUE )
ppm

Filename          = C:\Users\Koshino\Documen
Author            = delta
Experiment        = tocsy.jxp
Sample_Id         = 444-Namako-sample7-CD3OD
Solvent           = METHANOL-D3
Creation_Time     = 17-OCT-2011 15:06:59
Revision_Time     =  2-DEC-2012 13:53:34
Current_Time      =  2-DEC-2012 22:11:32

Comment           = Namako sample7 cd3od fgt
Data_Format       = 2D COMPLEX COMPLEX
Dim_Size          = 1638, 512
Dim_Title         = Proton Proton
Dim_Units         = [ppm] [ppm]
Dimensions        = X Y
Spectrometer      = DELTA2_NMR

Field_Strength    = 14.09636928[T] (600[MHz]
X_Acq_Duration    = 0.303104[s]
X_Domain          = 1H
X_Freq            = 600.1723046[MHz]
X_Offset          = 4[ppm]
X_Points          = 2048
X_Prescans        = 4
X_Resolution      = 3.29919764[Hz]
X_Sweep           = 6.75675676[kHz]
X_Sweep_Clipped   = 5.40540541[kHz]
Y_Domain          = 1H
Y_Freq            = 600.1723046[MHz]
Y_Offset          = 4[ppm]
Y_Points          = 256
Y_Prescans        = 0
Y_Resolution      = 21.10345759[Hz]
Y_Sweep           = 5.40248514[kHz]
Irr_Domain        = Proton
Irr_Freq          = 600.1723046[MHz]
Irr_Offset        = 5[ppm]
Tri_Domain        = Proton
Tri_Freq          = 600.1723046[MHz]
Tri_Offset        = 5[ppm]
Clipped           = FALSE
Scans             = 4
Total_Scans       = 1024

Relaxation_Delay  = 2.03[s]
Recvr_Gain        = 38
Temp_Get          = 25[dC]
Mix_Time          = 45[ms]
X_Acq_Time        = 0.303104[s]
X_Atn             = 3.4[dB]
X_Pulse           = 13[us]
X_Spinlock_Atn    = 9.4[dB]
X_Spinlock_Pulse  = 26[us]
Y_Acq_Time        = 47.3856[ms]
Y_P1_Correction   = 180
```

Fig. E   NOESY sepctrum of F7 fraction.

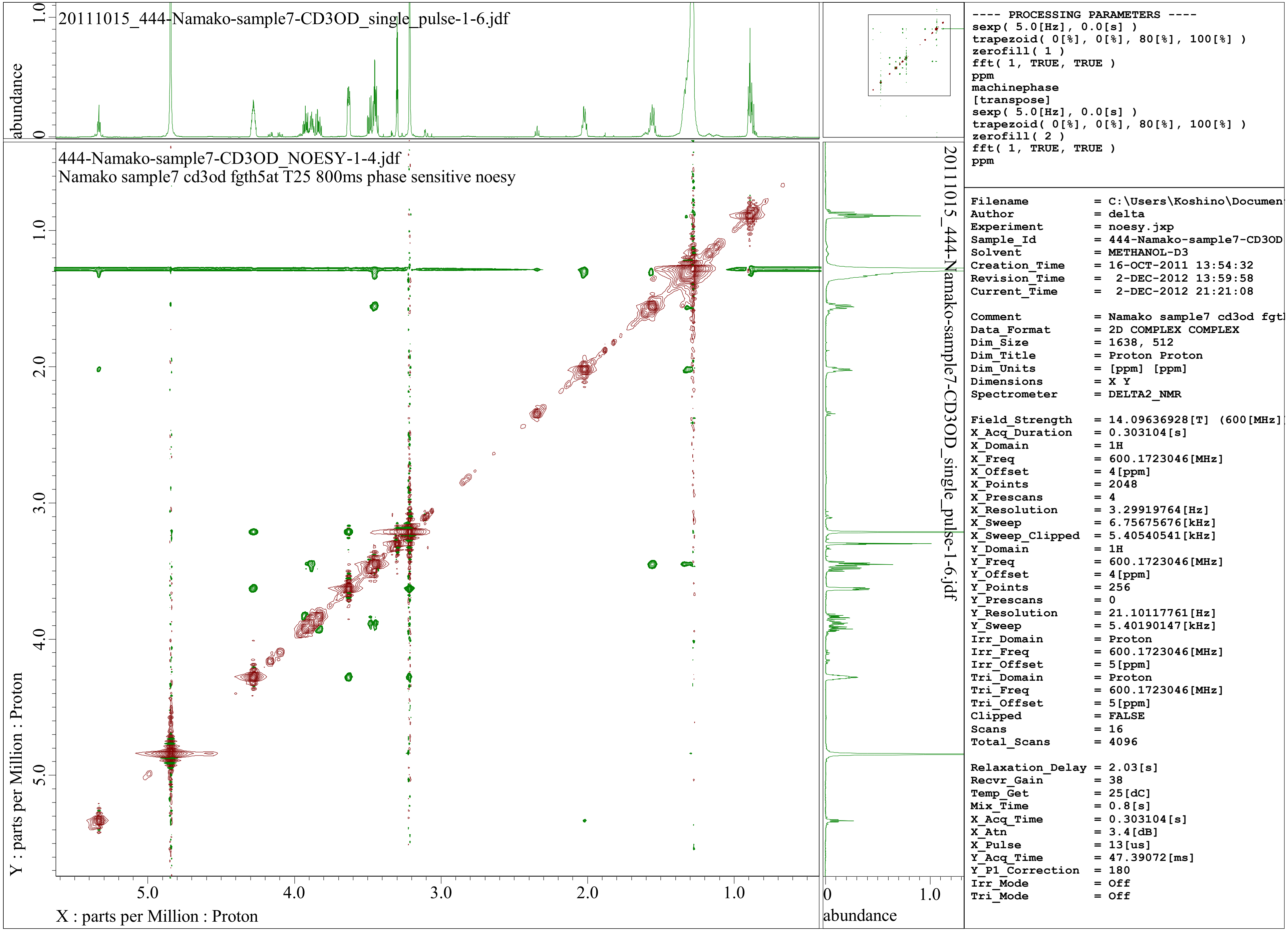

Fig. F HSQC spectrum of F7 fraction.

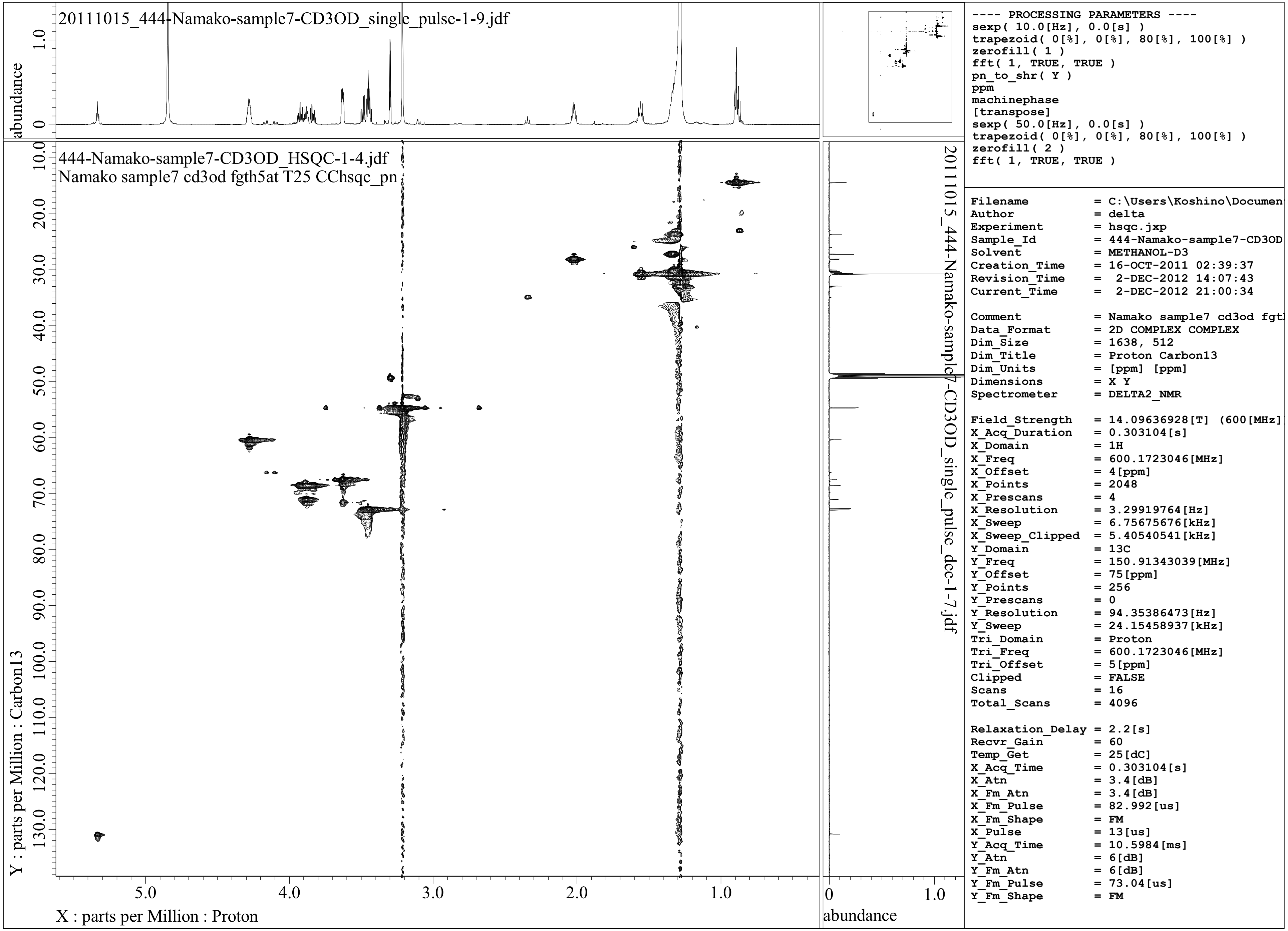

Fig. G HSQC-TOCSY spectrum of F7 fraction.

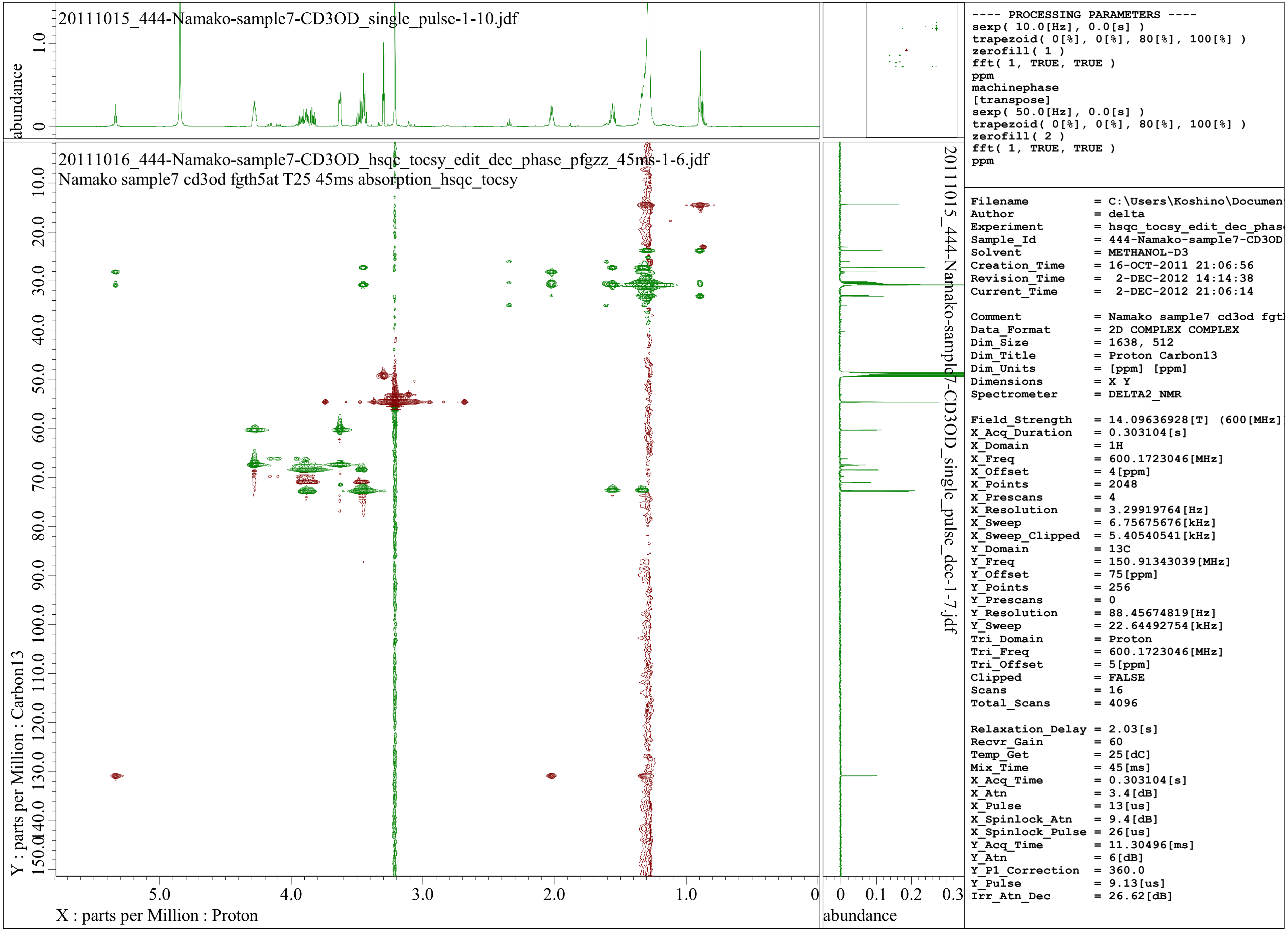

Fig. H ^{1}H-^{13}C HMBC spectrum of F7 fraction.

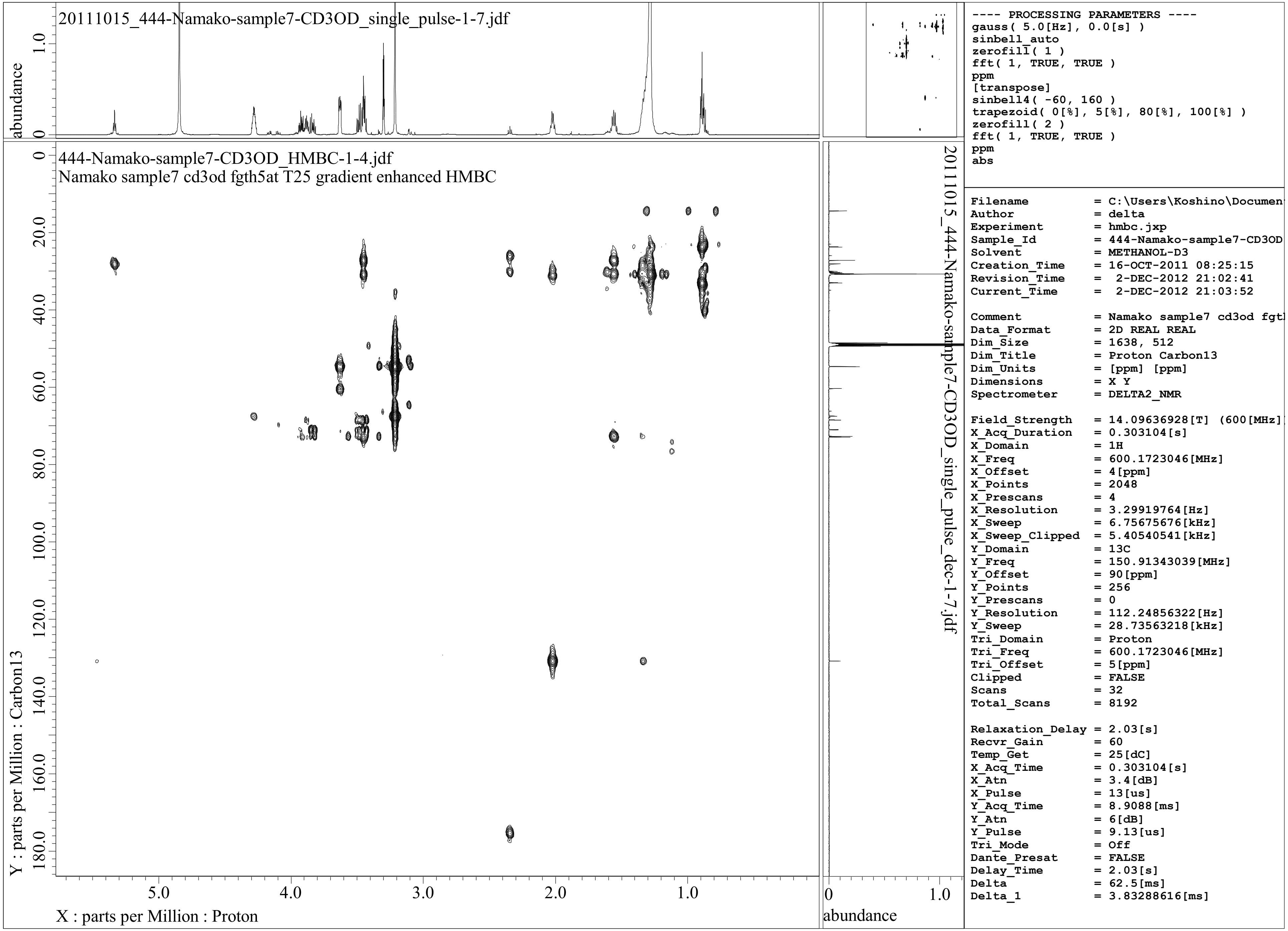

Fig. I ^{1}H-^{31}P HMBC spectrum of F7 fraction

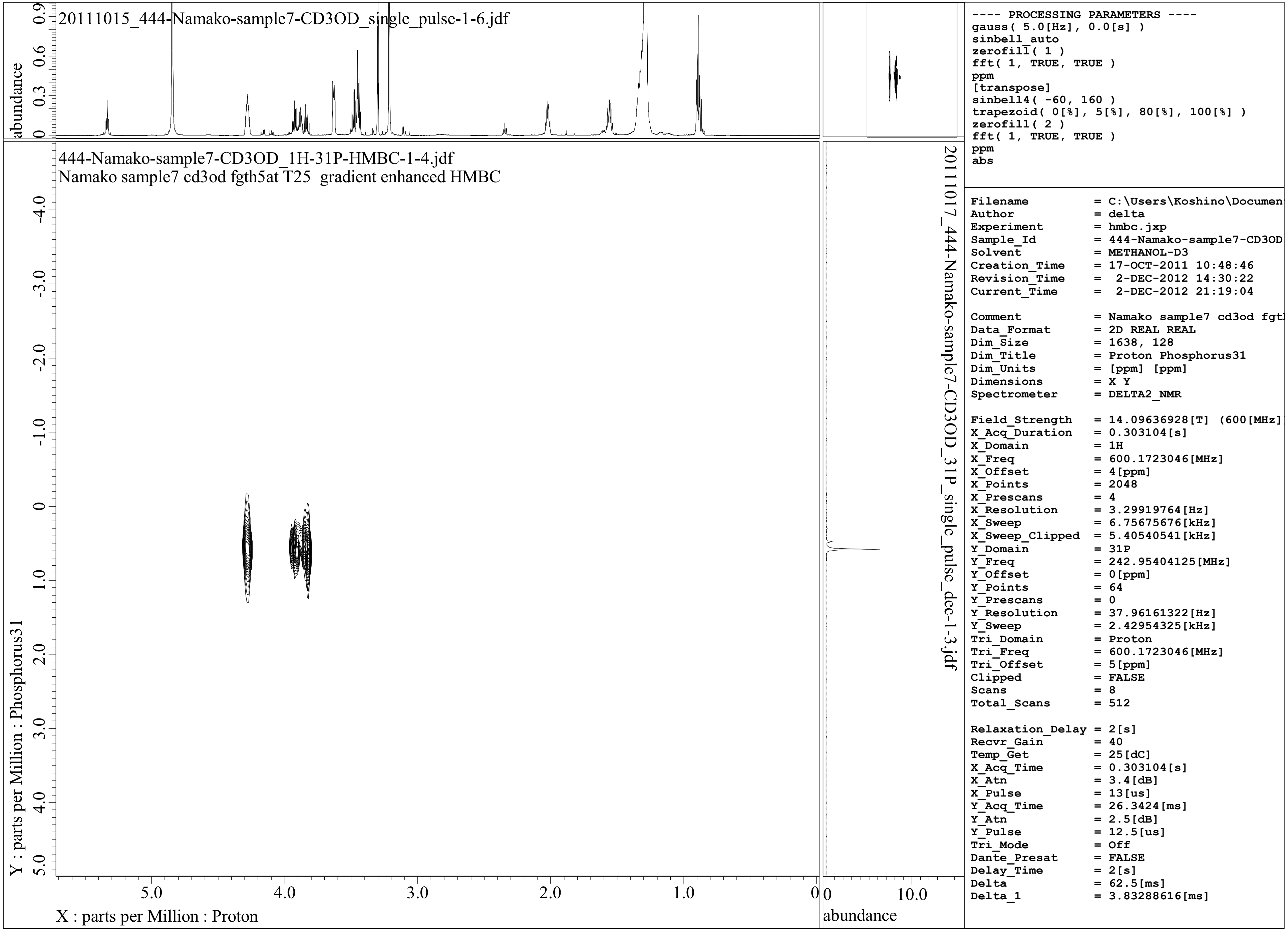

Fig. J Base peak ion chromatograms and mass chromatograms on LC/TOF-MS.

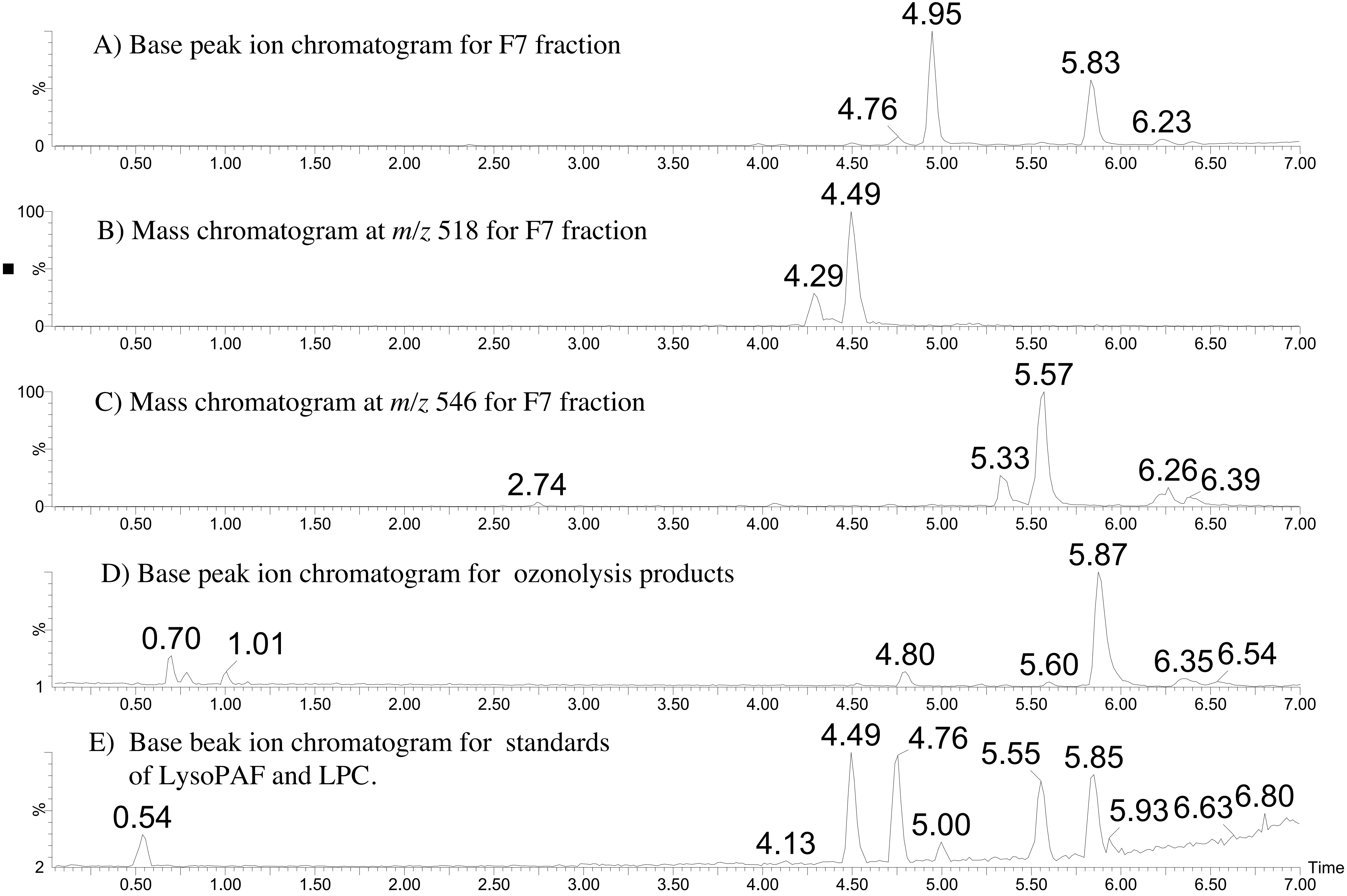

Fig. K Mass spctrum for RT 5.83 of F7 fraction, Lyso-PAF C18:0 (**1**).

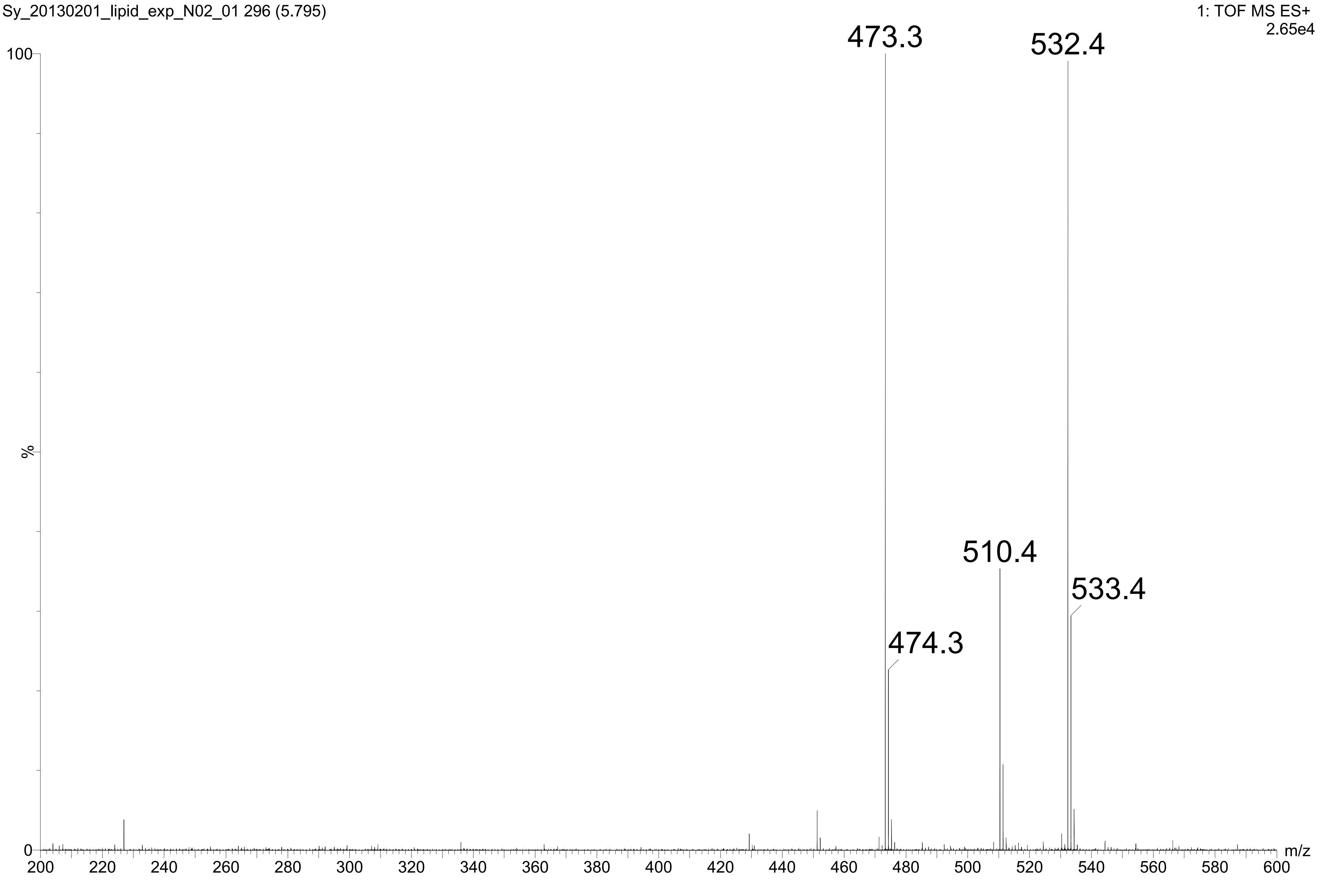

Fig. L Mass Spectrum for RT 4.95 of F7 fraction, Lyso-PAF (11*Z*)-C18:1 (**2**).

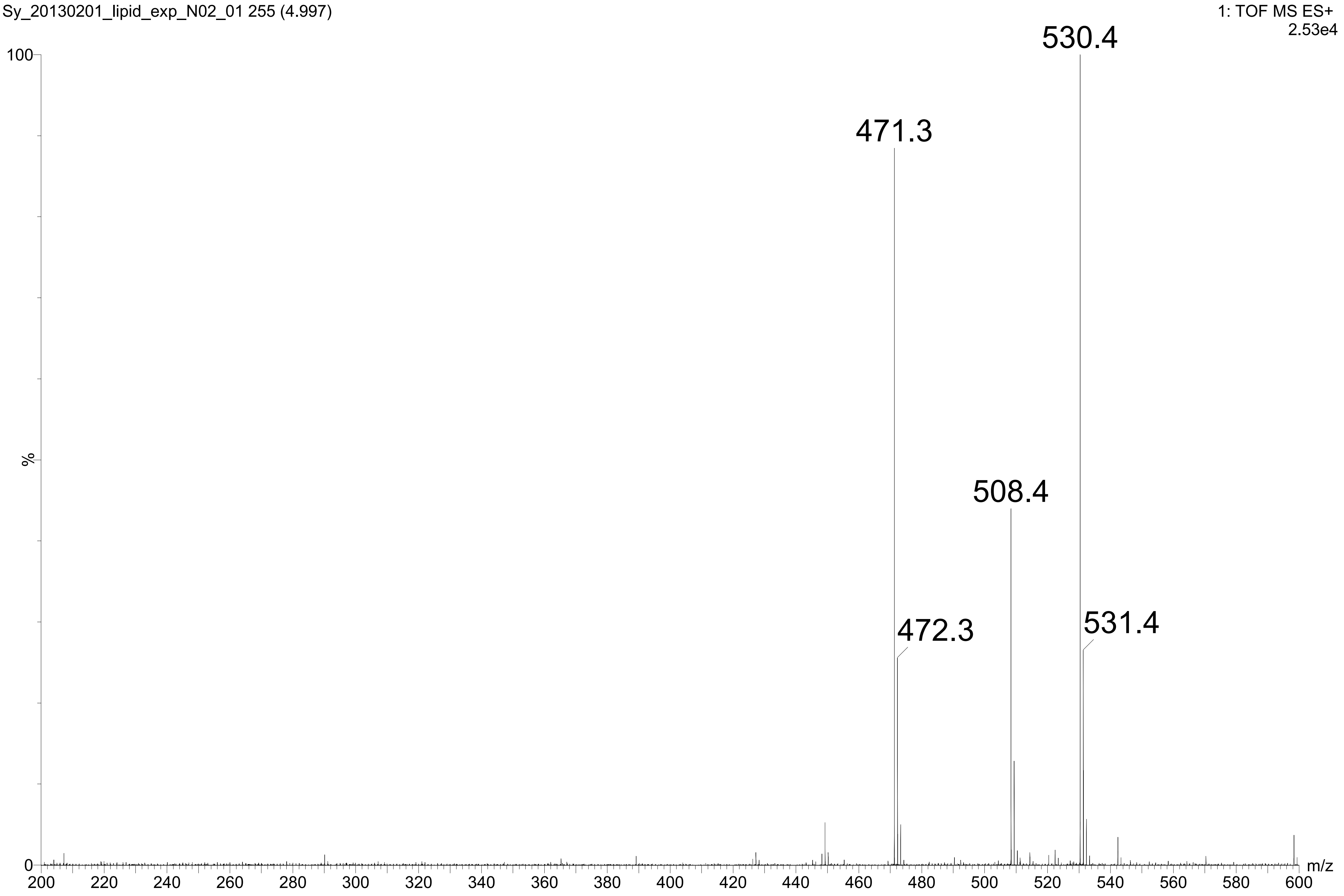

Fig. M Mass spectrum for RT 5.57 of F7 fraction, LPC C18:0 (**3**).

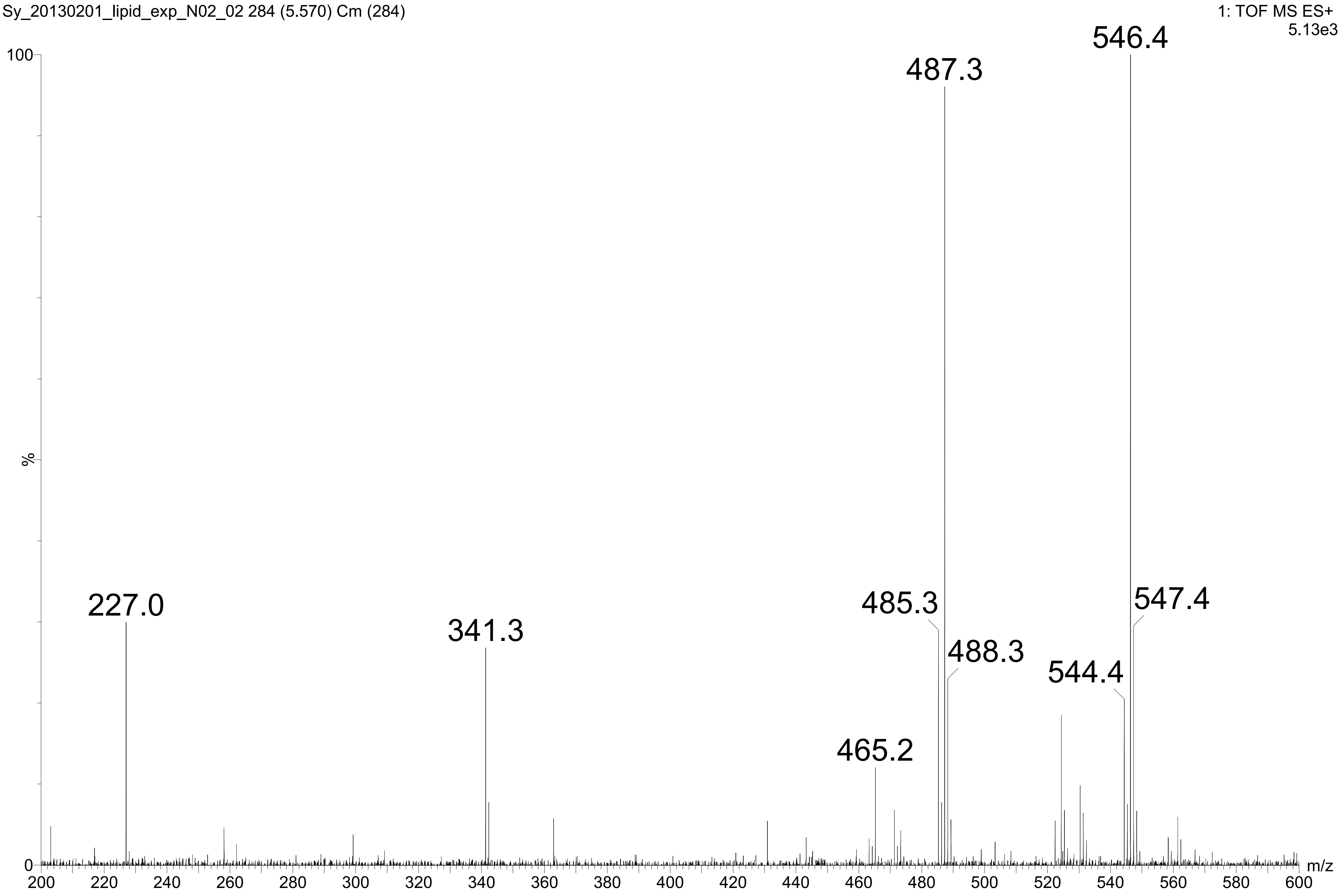

Fig. N Mass spectrum for RT 4.49 of F7 fraction, LPC C16:0 (**4**).

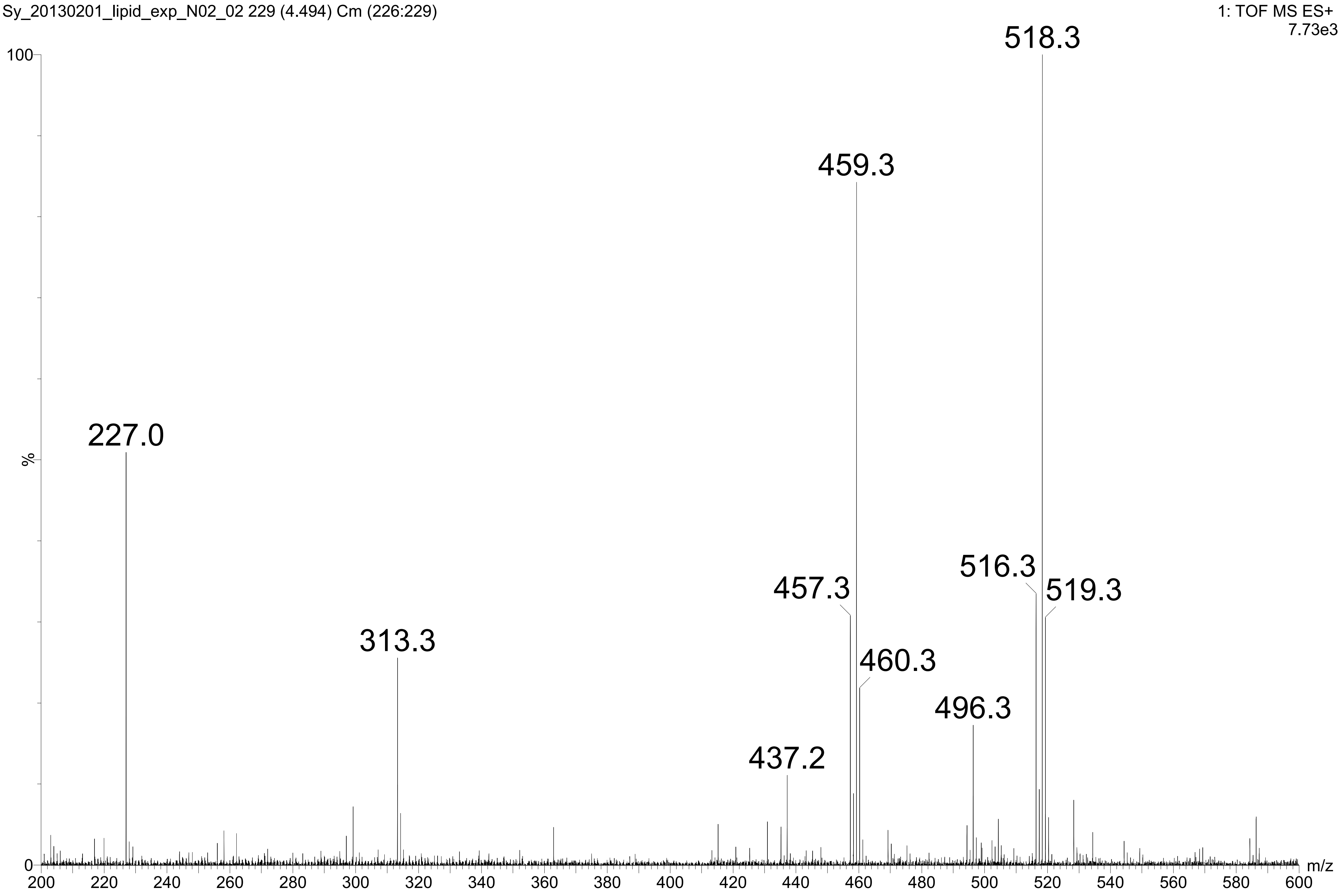

Fig. O Mass spectrum for RT 4.76 of F7 fraction, Lyso-PAF C16:0 (**5**).

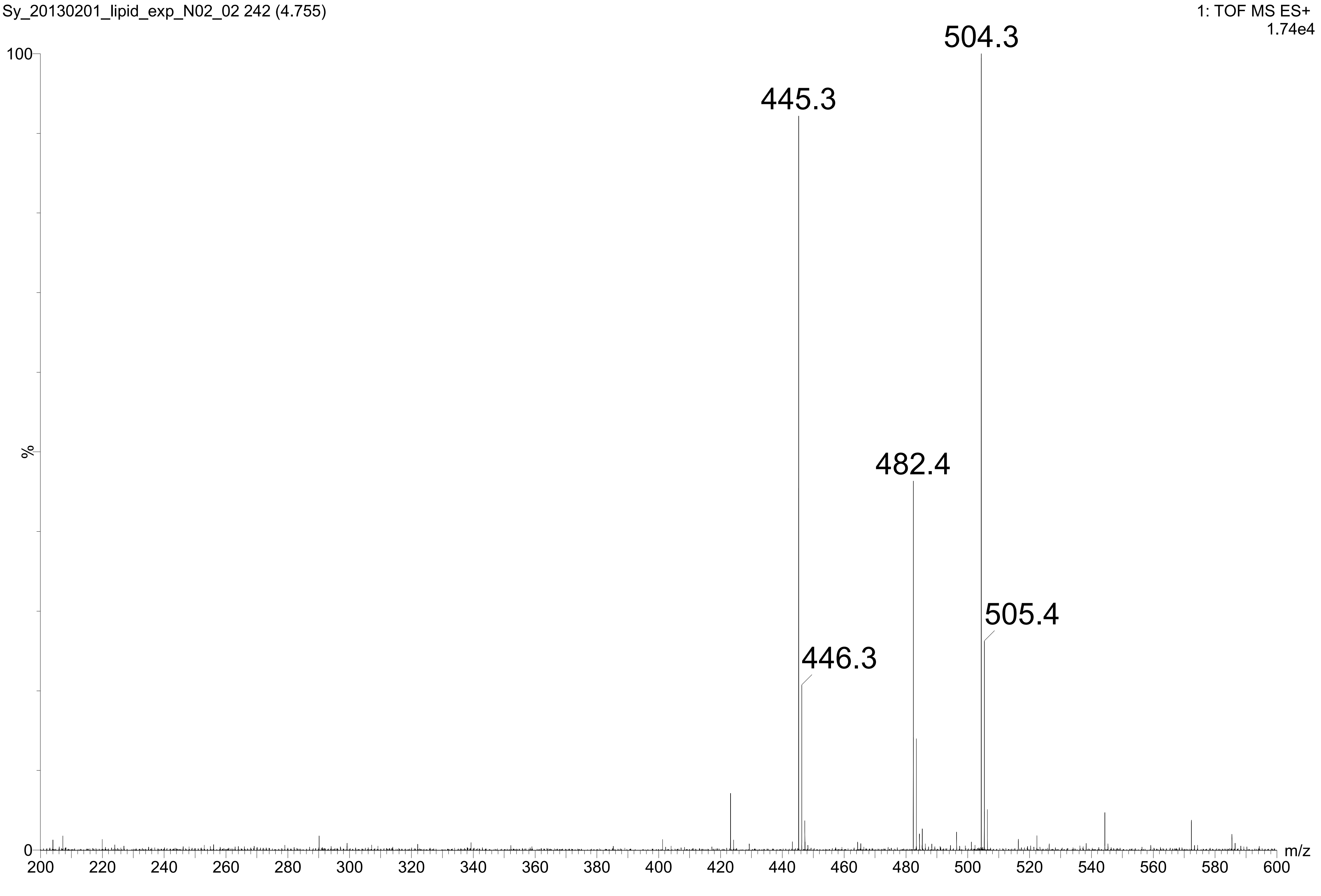

Fig. P   Mass spectrum for RT 6.63 of F7 fraction, Lyso-PAF 17'-Methyl-C18:0 (**6**).

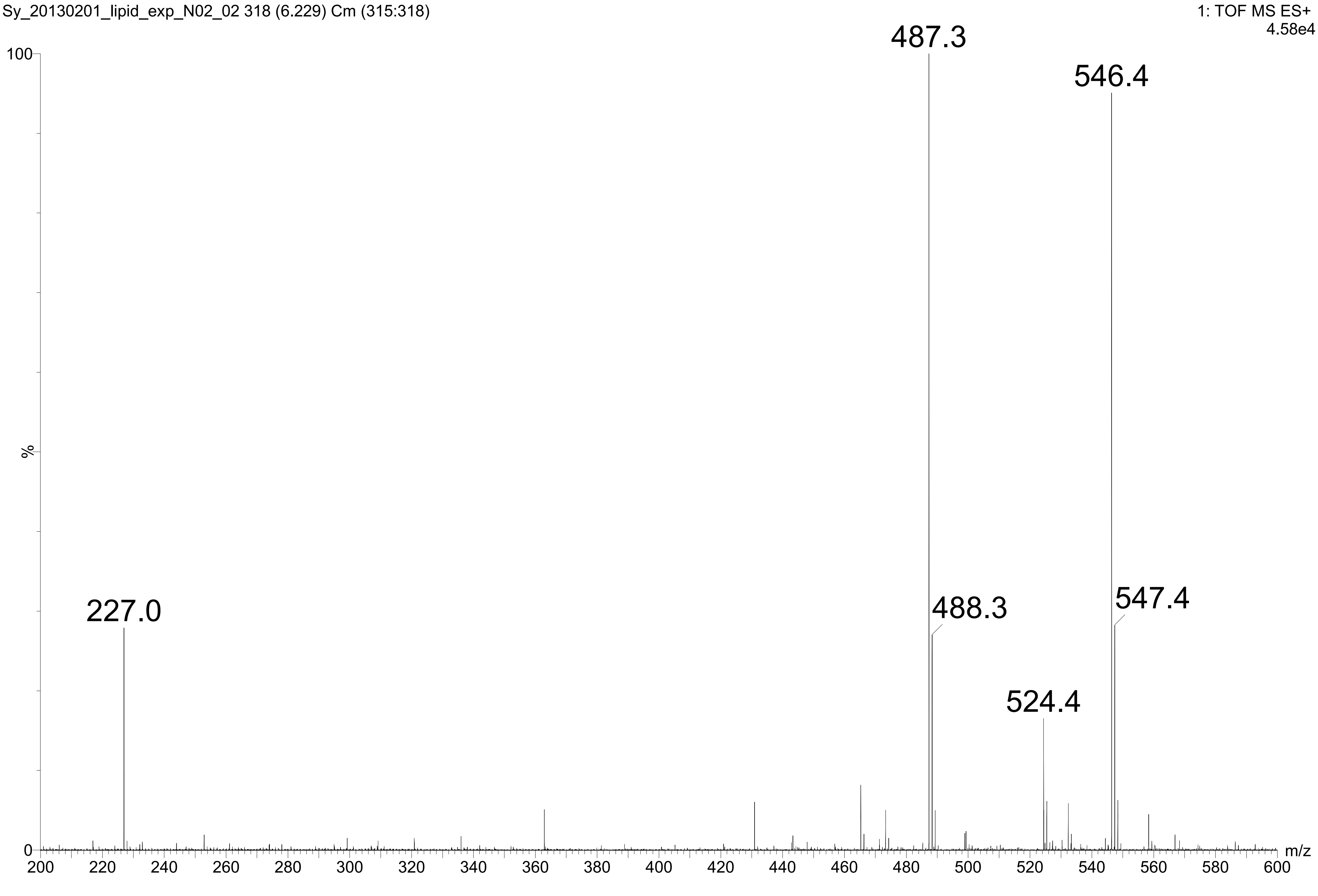

Fig. Q   Mass spectrum for Lyso-PAF C18:0 (1) of a standard mixture

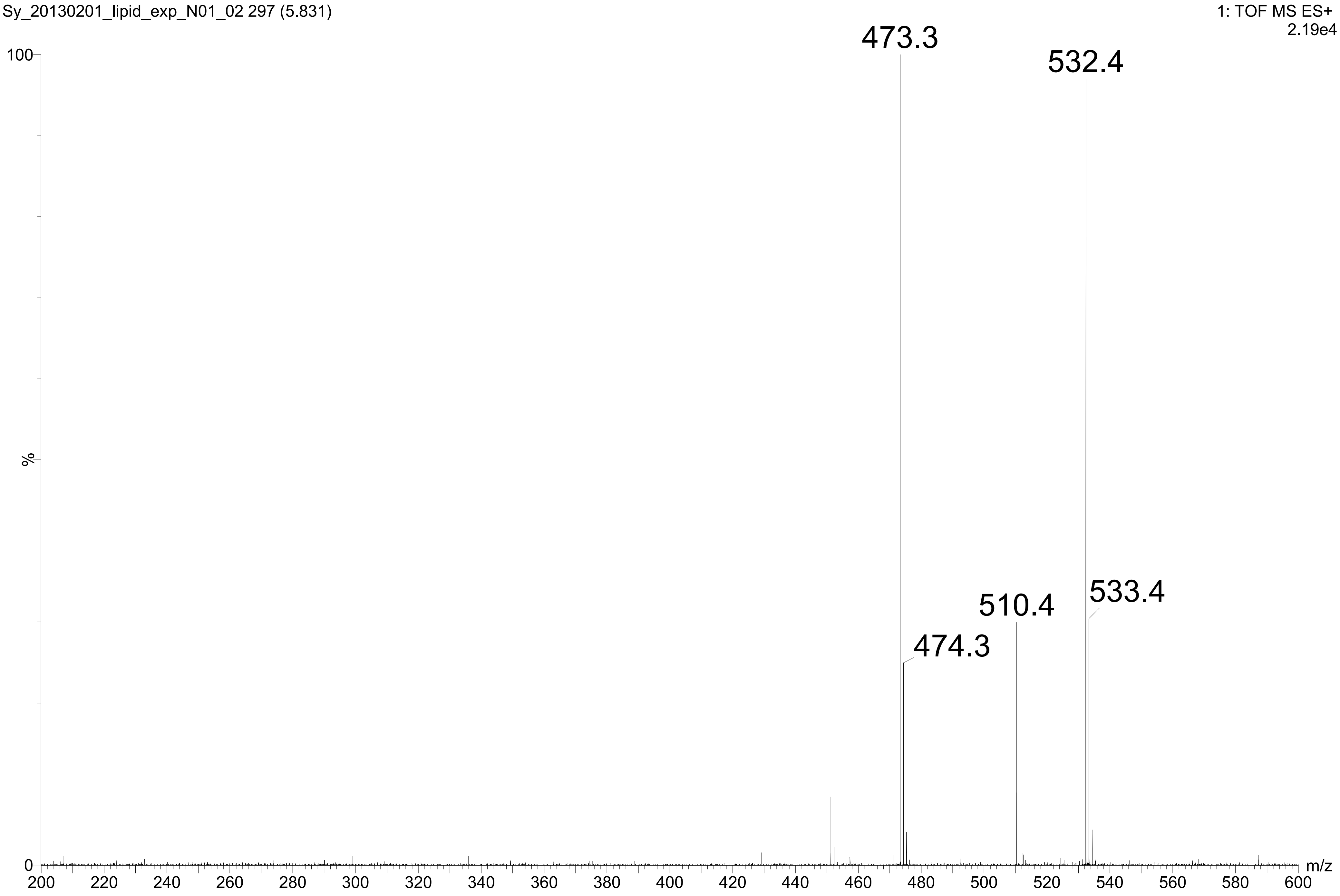

Fig. R Mass spectrum for Lyso-PAF (9Z)-C18:1 of a standard mixture.

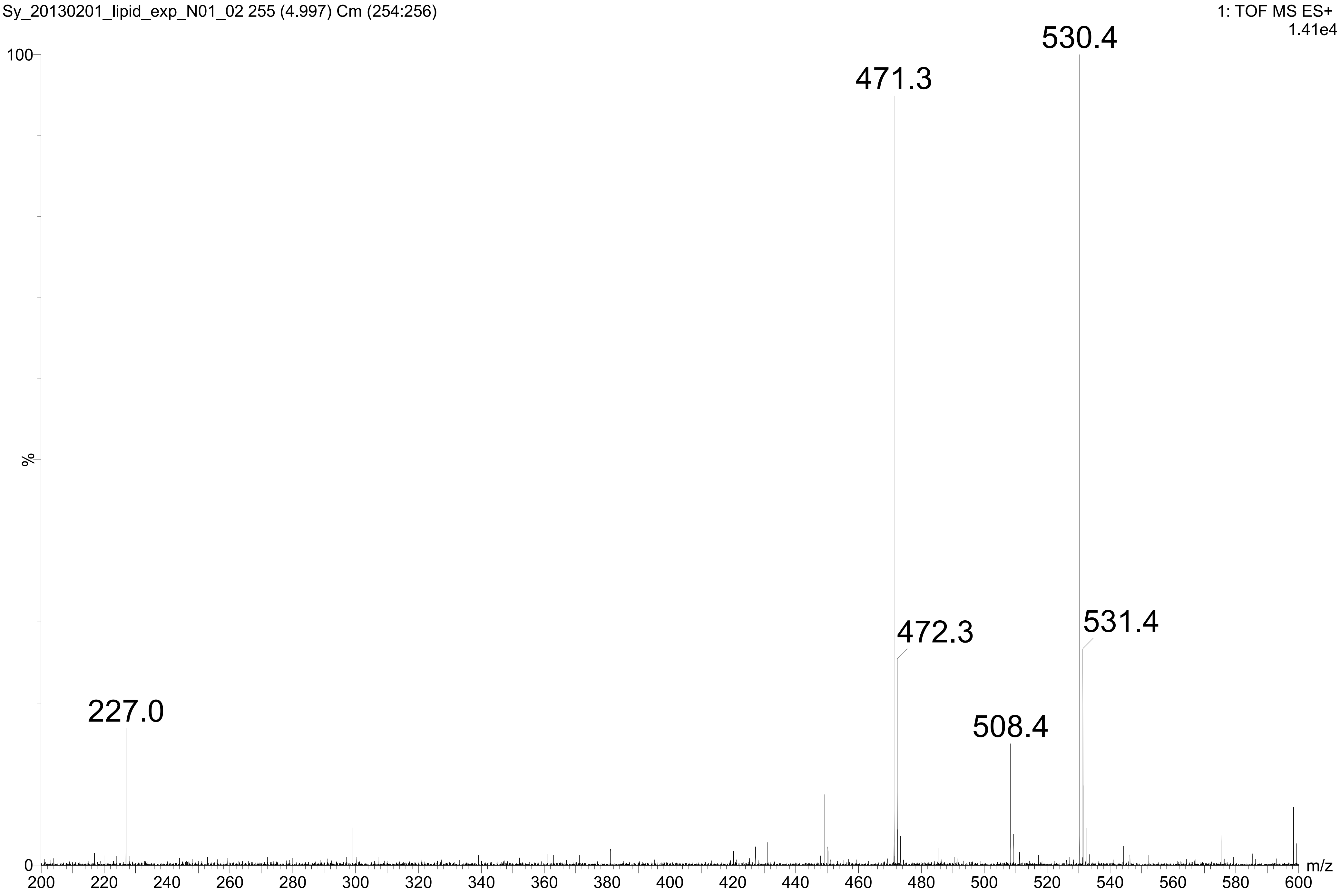

Fig. S Mass spectrum for LPC C18:0 (**3**) of a standard mixture.

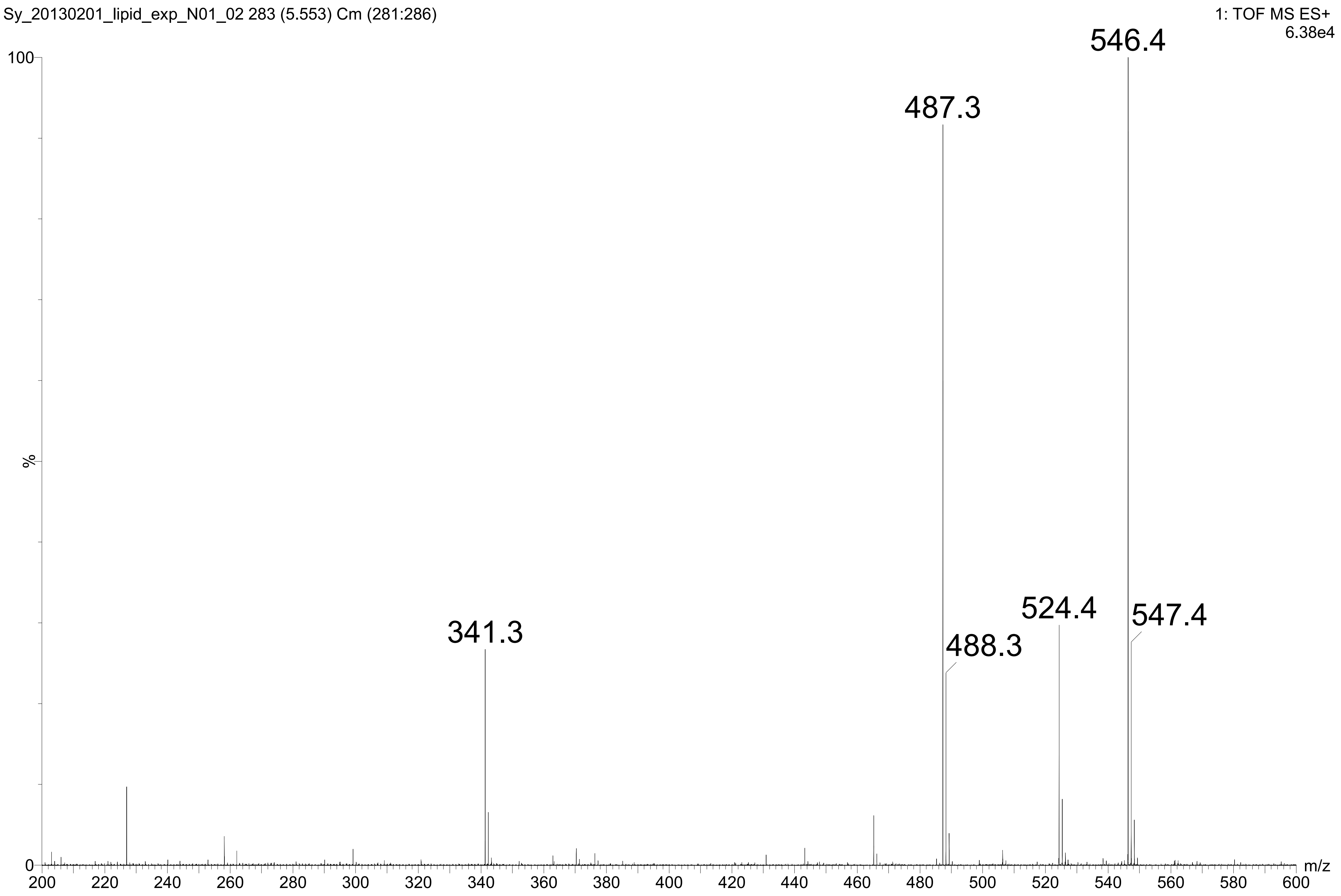

Fig. T Mass spectrum for LPC C16:0 (**4**) of a standard mixture.

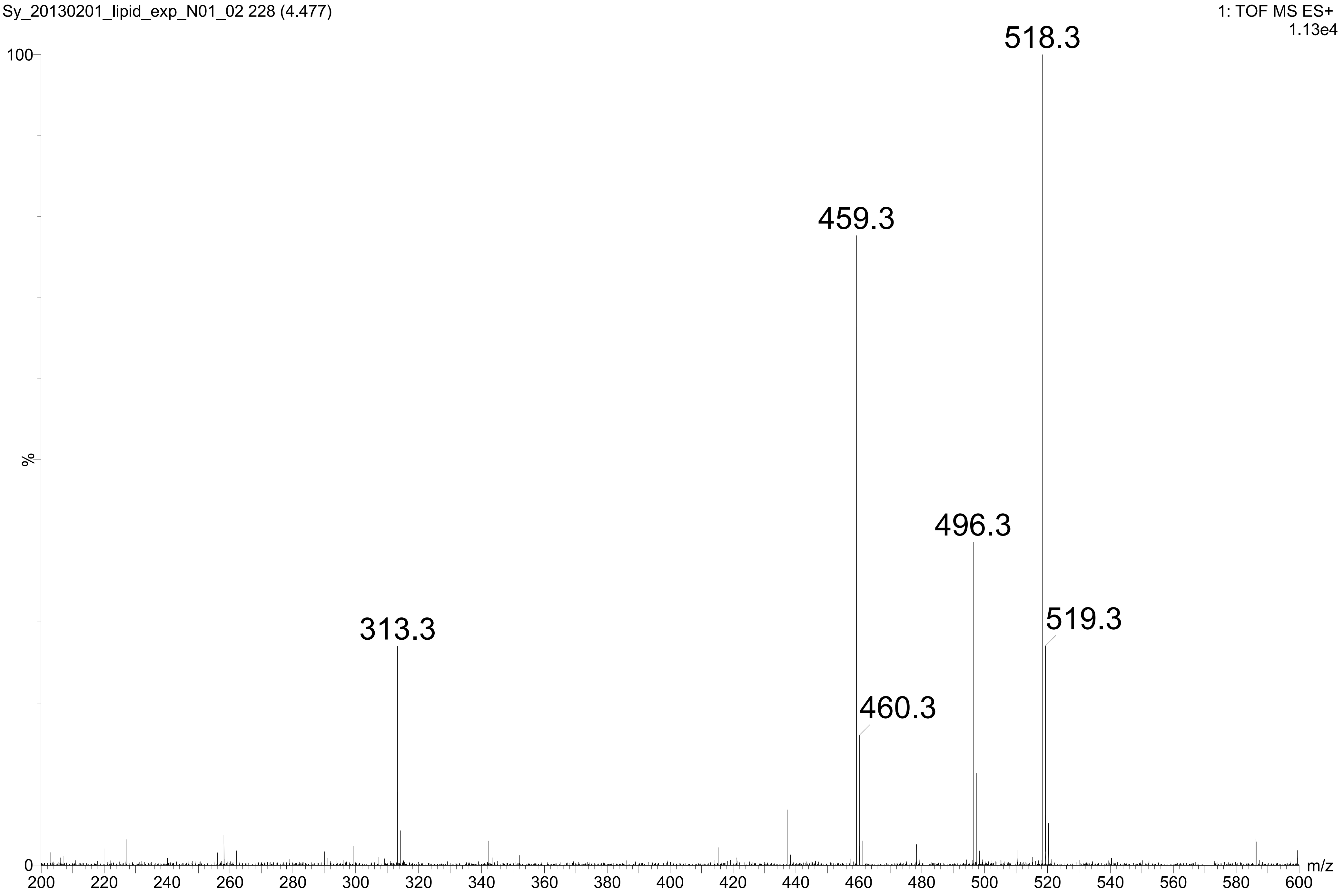

Fig. U Mass spectrum for LPC (9*Z*)-C18:1 of a standard mixture.

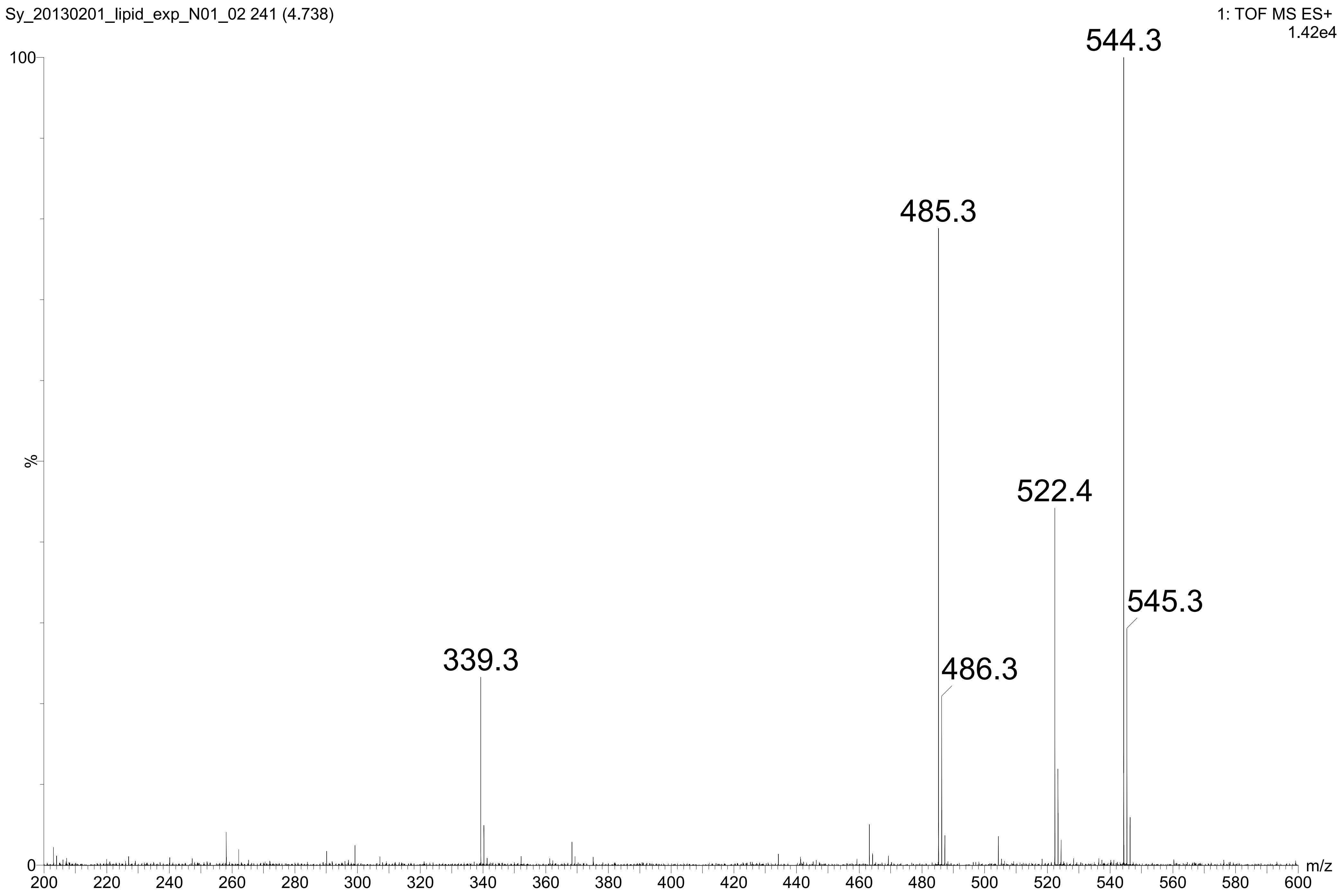

Fig. V Mass spectrum for RT 0.70 of ozonolysis products, C11-carboxylic acid (**7**).

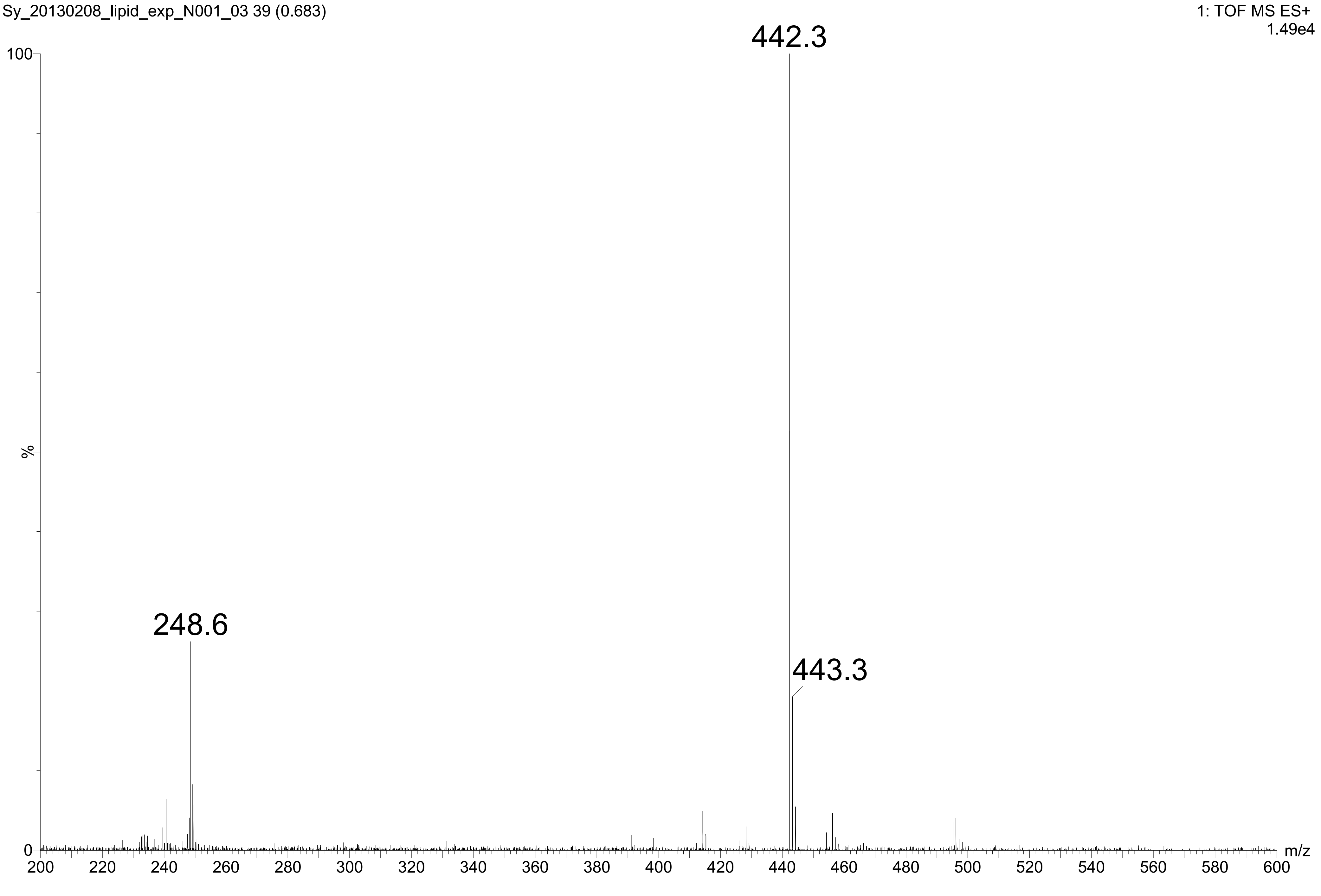

Fig. W Mass spectrum for RT 0.78 of ozonolysis products, C11-aldehyde (**8**).

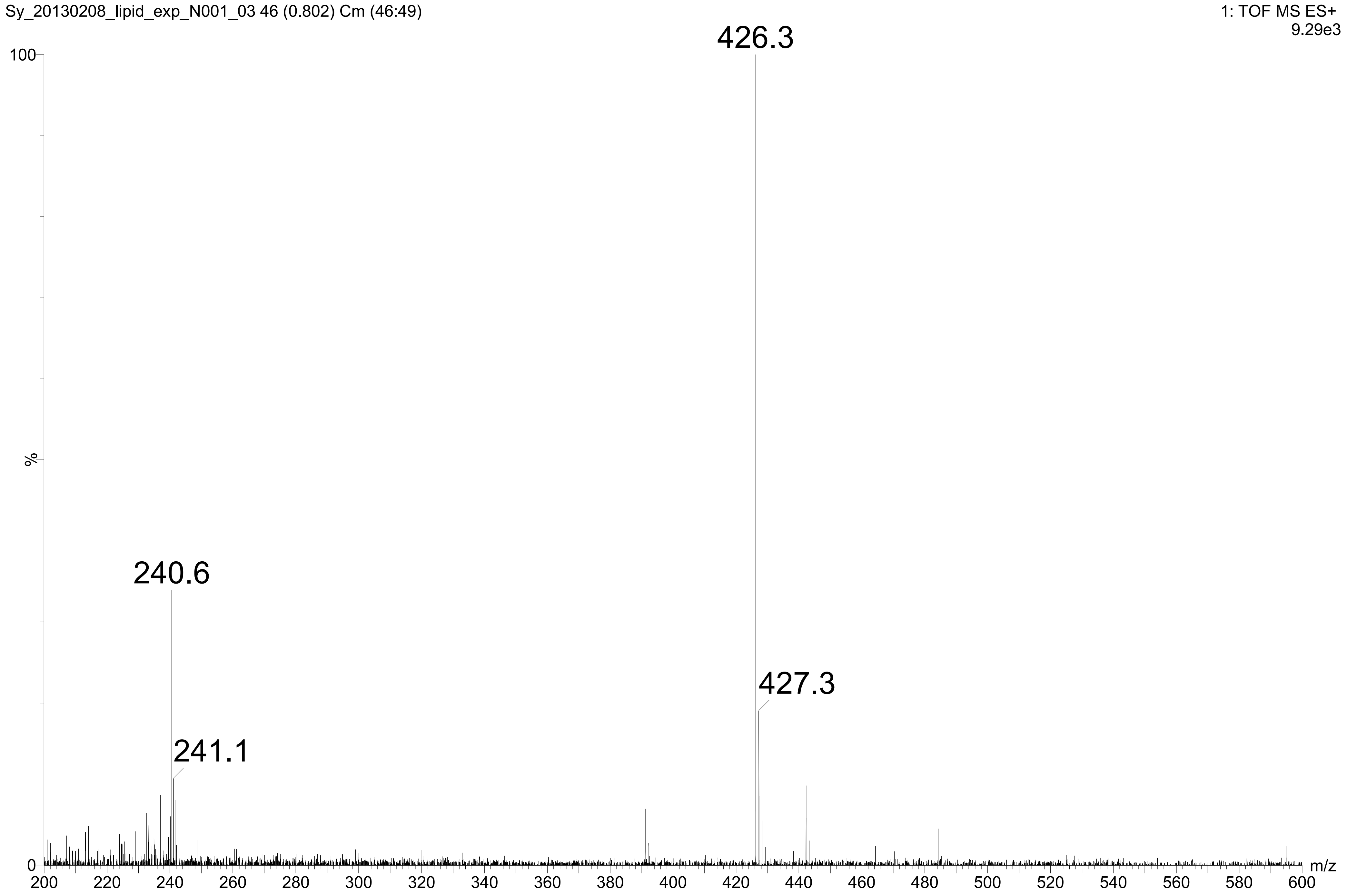

Fig. X Mass spectrum for RT 1.01 of ozonolysis products, C11-*d6*-dimethyl acetal (**9**).

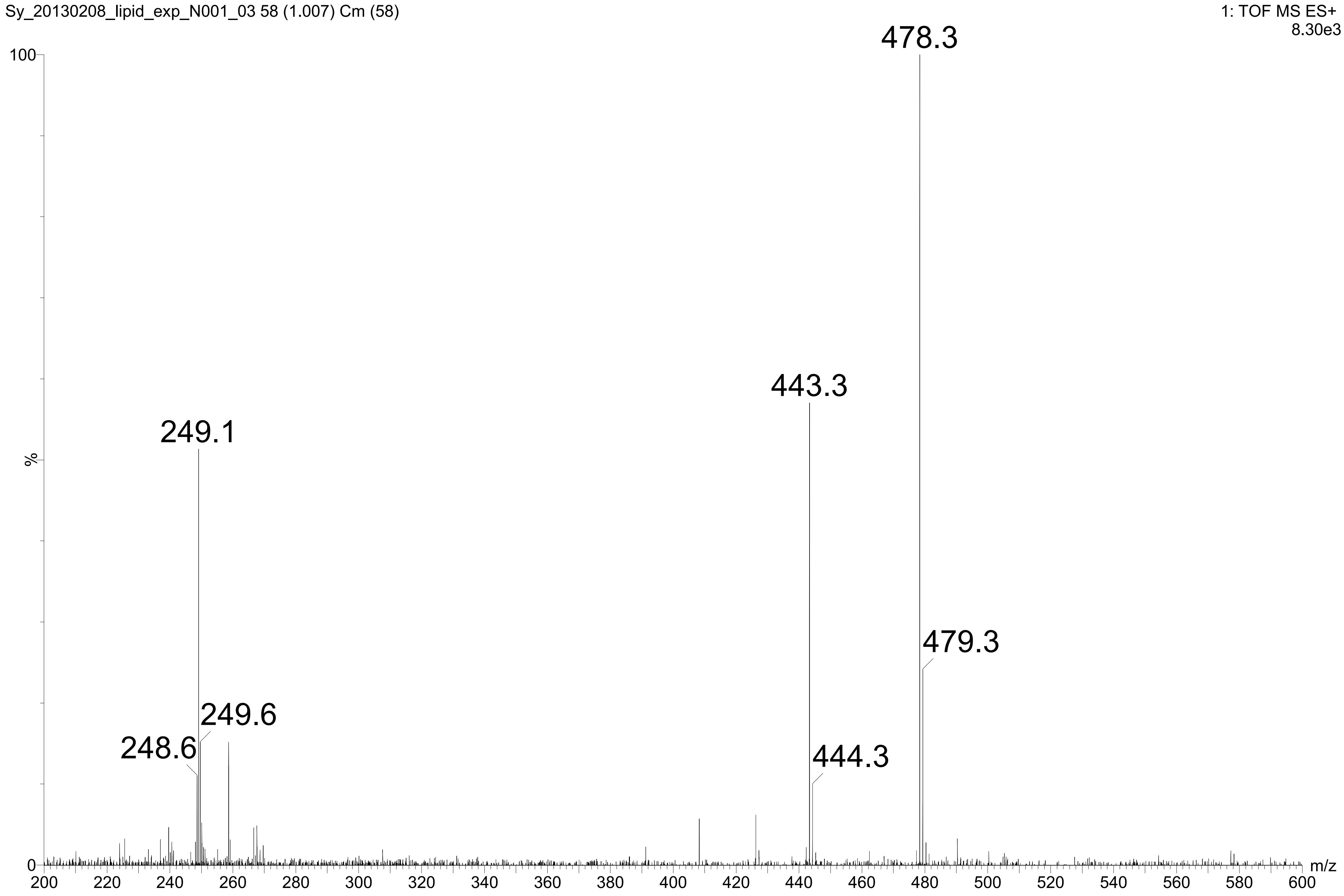

Supplement: S1 File — Fig A 1H NMR spectra of F7 fraction, and authentic Lyso-PAF (9Z)-C18:1. Fig B 13C NMR spectra of F7 fraction, and authentic Lyso-PAF (9Z)-C18:1 and cis-5-dodecenoic acid. Fig C DQF-COSY spectrum of F7 fraction. Fig D TOCSY spectrum of F7 fraction. Fig E NOESY spectrum of F7 fraction. Fig F HSQC spectrum of F7 fraction. Fig G HSQC-TOCSY spectrum of F7 fraction. Fig H 1H-13C HMBC spectrum of F7 fraction. Fig I 1H-31P HMBC spectrum of F7 fraction. Fig J Base peak ion chromatograms and mass chromatograms on LC/TOF-MS. Fig K Mass spectrum for RT 5.83 of F7 fraction, Lyso-PAF C18:0 (1). Fig L Mass spectrum for RT 4.95 of F7 fraction, Lyso-PAF (11Z)-C18:1 (2). Fig M Mass spectrum for RT 5.57 of F7 fraction, LPC C18:0 (3) Fig N Mass spectrum for RT 4.49 of F7 fraction, LPC C16:0 (4) Fig O Mass spectrum for RT 4.76 of F7 fraction, Lyso-PAF C16:0 (5). Fig P Mass spectrum for RT 6.23 of F7 fraction, Lyso-PAF 17’- Methyl-C18:0 (6). Fig Q Mass spectrum for Lyso-PAF C18:0 (1) of a standard mixture. Fig R Mass spectrum for Lyso-PAF (9Z)-C18:1 of a standard mixture. Fig S Mass spectrum for LPC C18:0 (3) of a standard mixture. Fig T Mass spectrum for LPC C16:0 (4) of a standard mixture. Fig U Mass spectrum for LPC (9Z)-C18:1 of a standard mixture. Fig V Mass spectrum for RT 0.70 of ozonolysis products, C11-carboxylic acid (7). Fig W Mass spectrum for RT 0.78 of ozonolysis products, C11-aldehyde (8). Fig X Mass spectrum for RT 1.01 of ozonolysis products, C11-d6-dimethyl acetal (9). (PDF) [file pone.0135701.s001.pdf]
